# Discovery and preclinical evaluation of anti-miR-17 oligonucleotide RGLS4326 for the treatment of polycystic kidney disease

**DOI:** 10.1038/s41467-019-11918-y

**Published:** 2019-09-12

**Authors:** Edmund C. Lee, Tania Valencia, Charles Allerson, Annelie Schairer, Andrea Flaten, Matanel Yheskel, Kara Kersjes, Jian Li, Sole Gatto, Mandeep Takhar, Steven Lockton, Adam Pavlicek, Michael Kim, Tiffany Chu, Randy Soriano, Scott Davis, John R. Androsavich, Salma Sarwary, Tate Owen, Julia Kaplan, Kai Liu, Graham Jang, Steven Neben, Philip Bentley, Timothy Wright, Vishal Patel

**Affiliations:** 1 Regulus Therapeutics Inc., San Diego, CA 92121 USA; 2 Department of Internal Medicine and Division of Nephrology, University of Texas Southwestern Medical Center, Dallas, TX 75390 USA

**Keywords:** Pharmaceutics, miRNAs, Polycystic kidney disease

## Abstract

Autosomal dominant polycystic kidney disease (ADPKD), caused by mutations in either *PKD1* or *PKD2* genes, is one of the most common human monogenetic disorders and the leading genetic cause of end-stage renal disease. Unfortunately, treatment options for ADPKD are limited. Here we report the discovery and characterization of RGLS4326, a first-in-class, short oligonucleotide inhibitor of microRNA-17 (miR-17), as a potential treatment for ADPKD. RGLS4326 is discovered by screening a chemically diverse and rationally designed library of anti-miR-17 oligonucleotides for optimal pharmaceutical properties. RGLS4326 preferentially distributes to kidney and collecting duct-derived cysts, displaces miR-17 from translationally active polysomes, and de-represses multiple miR-17 mRNA targets including *Pkd1* and *Pkd2*. Importantly, RGLS4326 demonstrates a favorable preclinical safety profile and attenuates cyst growth in human in vitro ADPKD models and multiple PKD mouse models after subcutaneous administration. The preclinical characteristics of RGLS4326 support its clinical development as a disease-modifying treatment for ADPKD.

## Abstract (web summary)

Autosomal dominant polycystic kidney disease (ADPKD) is a leading genetic cause of end-stage renal disease with limited treatment options. Here the authors discover and characterize a microRNA inhibitor as a potential treatment for ADPKD.

## Introduction

ADPKD is characterized by slowly progressive, bilateral kidney enlargement due to numerous fluid-filled cysts^1–3^. ADPKD is caused by mutations in either *PKD1* or *PKD2* genes, where disruption of their normal functions leads to excessive proliferation of the renal tubular epithelium causing cyst formation. Over 12 million people worldwide have ADPKD, making it amongst the most commonly known monogenetic disorders. Fifty percent of ADPKD patients eventually develop end-stage renal disease (ESRD) by the age of 60, accounting for 10% and 5% of prevalent patients with ESRD in Europe and the United States, respectively. Unfortunately, treatment options for this life-threatening disorder are still limited and inadequate.

miRNAs are evolutionarily conserved, ~20 nucleotides-long (nt) non-coding RNAs that function as post-transcriptional inhibitors of gene expression. Watson-Crick base pairing between the seed sequence (located primarily in nucleotide (N) position N2-to-N8 at the 5′ end) of the miRNA and the complementary sequences (found mainly in the 3′ untranslated region (3′UTR)) of mRNAs results in translational repression and eventual degradation of the targeted mRNA transcripts^4–6^. Aberrant activation of miRNAs has been shown to promote the progression of multiple human diseases; therefore, miRNA inhibition has emerged as an attractive therapeutic strategy^7–10^. Anti-microRNA (anti-miRs) are single-stranded, chemically modified oligonucleotides designed to sterically inhibit miRNAs and de-repress downstream target mRNAs and encoded proteins. Indeed, long anti-miRs of 18–21 nt with full complementarity to specific pathogenic miRNAs have been shown to attenuate disease progression in both preclinical and clinical settings^11–16^.

We have recently shown that the miR-17 miRNAs family is upregulated in both human and murine forms of ADPKD, and their deletion or inhibition attenuates cyst growth in mouse PKD models^17–19^. The miR-17 family is derived from three polycistronic clusters: miR-17~92, miR-106a~363, and miR-106b~25 clusters^20^. Among the three, miR-17~92 is essential for embryonic development and is well-known for its role as an oncogene^21,22^. Germline knockout of miR-17~92 causes perinatal lethality with bone, heart, lung, and B cell maturation defects, while deletion of the miR-106a~363 or miR-106b~25 clusters does not produce any obvious abnormality^23^. Within the kidney, miR-17~92 deletion in mouse nephron progenitors impairs nephrogenesis^24^. However, the expression of these clusters declines with maturation, and accordingly, inducible deletion of miR-17~92 in adult mice does not impact their lifespan or general well-being, other than reduction in mature hematopoietic lineages^25^. Kidney-specific deletion of the miR-17~92 cluster also does not produce any appreciable defects in kidney morphology and histology^18^. Therefore, preferential targeting of the miR-17 family in the post-natal kidney is an attractive therapeutic approach to treat ADPKD.

Based on our previous experience with tool anti-miR-17 oligonucletides^17,26,27^ and known properties of chemically modified oligonucleotides^28–30^, we designed and screened for anti-miR-17 oligonucleotides with favorable pharmaceutical properties that could be used for human clinical testing. Here, we describe the discovery and preclinical evaluation of RGLS4326, a single-stranded, chemically modified, short oligonucleotide of 9-nt with full complementarity to the miR-17 seed sequence. RGLS4326 is designed to preferentially target the kidney and inhibit the pathologic functions of the miR-17 family of miRNAs in ADPKD. Importantly, we report that RGLS4326 reproducibly attenuates cyst growth in human ADPKD models in vitro and multiple PKD mouse models in vivo. Our studies support the clinical development of RGLS4326 for the treatment of ADPKD.

## Results

### Discovery of anti-miR-17 oligonucleotide RGLS4326

**Fig. 1:**
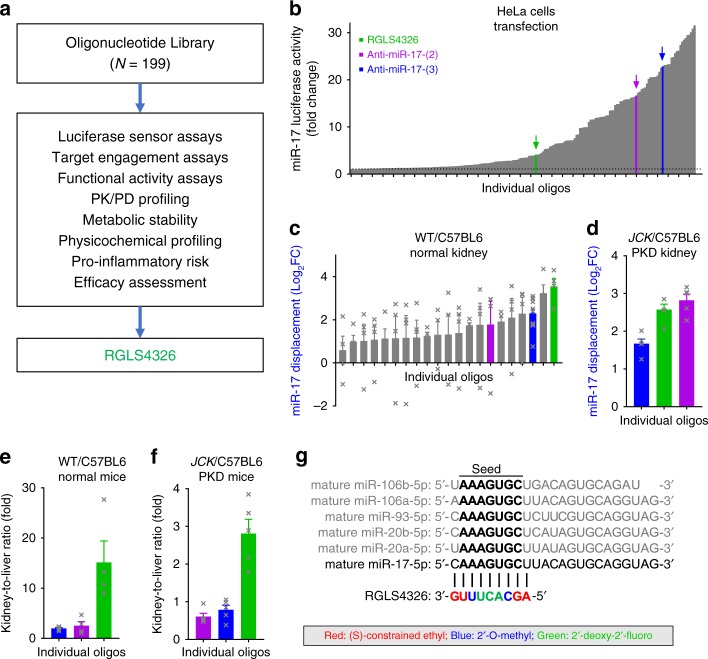
Discovery of RGLS4326, a chemically modified oligonucleotide inhibitor of miR-17. **a** Screening cascade used for the discovery of RGLS4326. **b** Over 190 anti-miR-17 oligonucleotides of diverse chemical designs were screened at 10 μM in a miR-17 HeLa cell luciferase assay and plotted in ascending order of potency (*n* = 1/oligonucleotides). Selected oligonucleotides including RGLS4326 (green) are highlighted for illustration purposes. **c** A subset of oligonucleotides was tested in WT/C57BL6 mice for their ability to engage and displace miR-17 in the kidney 7 days after a single 30 mg kg^−1^ SC dose (*n* = 4). **d** A smaller set of oligonucleotides were further tested in the *JCK*/C57BL6 PKD model for miR-17 target engagement (*n* = 5). **e**–**f** Preferential distribution to kidney over liver 7 days after a single 30 mg kg^−1^ SC dose of selected oligonucleotides in WT/C57BL6 (*n* = 4) and JCK/C57BL6 mice (*n* = 5) are shown. **g** Chemical modifications, base sequence, and corresponding complementarity to the miR-17 family of mature miRNAs for RGLS4326 is illustrated. Error bars represent standard error of means. Source data for Fig. 1**b**–**f** is provided in Source data files

We discovered RGLS4326 by screening a chemically diverse library of anti-miR-17 oligonucleotides for optimal pharmaceutical properties including potency, stability, safety, pharmacokinetic-pharmacodynamic profile, and ability to preferentially distribute to kidney and confer therapeutic efficacy following systemic administration (Fig. 1a). The library contained >190 oligonucleotides of different lengths, base sequences (complementary to varying positions against mature miR-17), and chemical modifications that were methodically varied to optimize for favorable pharmaceutical profiles. We first screened this library using a miR-17 luciferase sensor assay, where a luciferase reporter vector that contained two fully complementary miR-17 binding sequences in the 3′UTR of the luciferase gene was used. Briefly, HeLa cells were co-transfected with the luciferase reporter vector and an exogenous miR-17-expression vector that acted to repress the luciferase signal. Next, HeLa cells were individually treated with anti-miR-17 oligonucleotides from our library. Their ability to sequester miR-17 and de-repress the luciferase signal is plotted in ascending order of potency in Fig. 1b. A subset of these anti-miR-17 oligonucleotides with a wide range of chemical designs and in vitro potency against miR-17 were further characterized in normal mice (Fig. 1c) followed by mice with PKD (Fig. 1d). We assessed their ability to engage, inhibit, and displace miR-17 from the translationally active high molecular weight (HMW) polysomes^26^ in the kidney following subcutaneous (SC) administration. We also evaluated their propensity for preferential distribution to kidney over liver while remaining metabolically stable (Fig. 1e, f).

Screening of this library led to the discovery of RGLS4326, a 9-nt phosphorothioate backbone-modified oligonucleotide composed of (S)-constrained ethyl, 2′-O-methyl, and 2′-deoxy-2′-fluoro nucleoside sugar modifications with a base sequence complementary to the N1-to-N9 position of the mature miR-17 (Fig. 1g). RGLS4326 preferentially distributed to both normal and polycystic kidney, and potently displaced miR-17 from translationally active polysomes in vivo. RGLS4326 was metabolically stable in mouse, monkey, and human tissue lysates and in mouse kidney and liver tissues following SC administration (Supplementary Table 1) and was selected for further evaluation.

### RGLS4326 inhibits miR-17 and derepresses direct mRNA targets

**Fig. 2:**
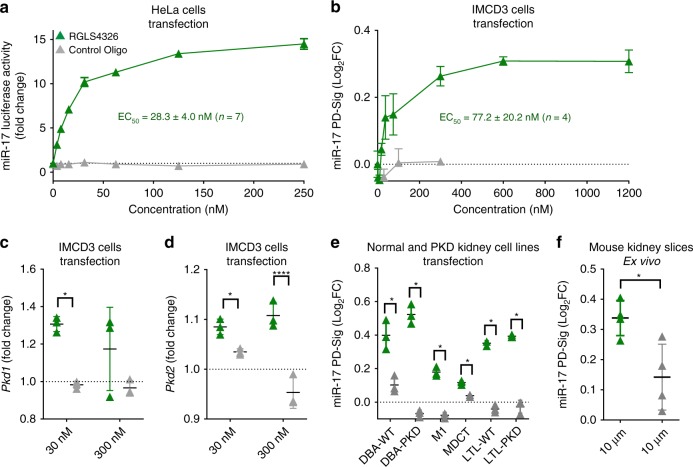
RGLS4326 inhibits miR-17 and de-represses direct miR-17 targets. **a** RGLS4326 (green triangle) dose-responsively inhibits miR-17 in HeLa cell luciferase assay 24 h after transfection, with an EC_50_ value of 28.3 ± 4.0 nM (mean ± standard deviation, *n* = 7 independent experiments). **b** RGLS4326 dose-responsively de-represses multiple miR-17 target genes (as measured by miR-17 PD-Sig) in mouse IMCD3 cells 24 h after transfection, with an EC_50_ value of 77.2 ± 20.2 nM (*n* = 4 independent experiments). **c**–**d** RGLS4326 treatment results in de-repression of the direct miR-17 target genes, *Pkd1* and *Pkd2* in IMCD3 cells (*n* = 3). **e** RGLS4326, but not control oligo, functionally inhibited miR-17 and de-repressed miR-17 PD-Sig in six cell lines derived from normal (DBA-WT, M1, MDCT, LTL-WT) or PKD (DBA-PKD and LTL-PKD) mouse kidneys after 24 h treatment by transfection at 30 nM (*n* = 3) and in **f** normal mouse kidney tissue slice culture after 72 h ex vivo incubation at 10 μM (*n* = 4). Control oligo (grey triangle) containing the same chemical-modification, length, and design as RGLS4326, but different base pair sequence, was used as a negative control. Error bars represent standard deviations. *p < 0.05, ****p < 0.0001. One-way ANOVA, Dunnette’s multiple comparison test. Source data for Fig. 2**a**–**f** is provided in Source data files

We first validated our initial screening results and performed dose-response analysis. RGLS4326 inhibited miR-17 function and de-repressed miR-17 luciferase sensor activity in HeLa cells with an EC_50_ value of 28.3 ± 4.0 nM (Fig. 2a). As anticipated from its sequence complementarity to the miR-17 family seed region, RGLS4326 also de-repressed luciferase sensor activity of other miR-17 family members, including miR-20b, miR-93, and miR-106a (Supplementary Fig. 1A–D), but not unrelated miRNAs such as miR-33b (Supplementary Fig. 1E).

In addition to the commonly used luciferase sensor assays, functional inhibition of miRNA is often assessed by measuring de-repression of individual downstream target genes. However, changes in individual miRNA target genes are typically small, variable, and largely cell-type dependent. To circumvent this issue, we developed a mouse miR-17 Pharmacodynamic-Signature (miR-17 PD-Sig), which consists of the expression of 18 unique miR-17 target genes normalized by six reference housekeeping genes, to provide an unbiased and comprehensive assessment of miR-17 activity. Briefly, four different mouse cell lines (of both kidney and non-kidney origins) were transfected with mock, a chemically diverse set of tool anti-miR-17 oligos, or a tool miR-17-mimic; and whole-transcriptome RNA-sequencing was performed. Direct miR-17 target genes (containing 3′UTR sequences complementary to miR-17 family seed) whose expression increased after anti-miR-17 treatment but decreased after miR-17-mimic treatment were ranked, filtered for non-redundancy, and selected as candidate genes (Supplementary Fig. 2A). These candidate genes were further validated by qPCR in one additional non-kidney cell line before the final top 18 ranked genes were chosen (Supplementary Fig. 2B–C). The resultant mouse miR-17 PD-Sig score is the calculated average of the 18 genes’ individual log_2_ fold changes (normalized by six housekeeping genes) compared to mock. Indeed, as exemplified in Supplementary Fig. 2D, our miR-17 PD-Sig can be used to comprehensively and unbiasedly assess miR-17 activity within a dynamic range that allows for rank-ordering of both anti-miR-17 and miR-17 mimic of interest.

We next utilized this mouse miR-17 PD-Sig to evaluate the activity of RGLS4326. RGLS4326 treatment inhibited miR-17 function in kidney collecting duct cells in culture as measured by miR-17 PD-Sig, with an EC_50_ value of 77.2 ± 20.2 nM (Fig. 2b). The direct miR-17 target genes *Pkd1* and *Pkd2*, which are central to ADPKD pathogenesis^2^ were also de-repressed (Fig. 2c, d). De-repression of miR-17 PD-Sig after RGLS4326 treatment was not restricted to collecting duct cells, as the same was also observed in six different mouse kidney cell lines of proximal tubule, distal tubule and collecting duct origins derived from normal and PKD mouse kidneys (Fig. 2e), and a mouse kidney slice culture ex vivo (Fig. 2f).

As previously demonstrated, functional inhibition of miR-17 can also be assessed by measuring the amount of miR-17 displaced from the translationally active HMW polysomes by miRNA polysome shift assay^26^ following anti-miR-17 treatment. We therefore evaluated miR-17 PD-Sig as readout of RGLS4326 activity in a head-to-head comparison with the miRNA polysome shift assay. As shown in Supplementary Fig. 3, we observed a positive and dose-dependent correlation between the displacement of miR-17 and de-repression of miR-17 PD-sig after RGLS4326 treatment. In contrast, treatment with control oligonucleotides had no effect. Taken together, our comprehensive screening cascade has enabled the discovery of RGLS4326 that engages and displaces miR-17 from translationally actively HMW polysomes thereby de-repressesing direct miR-17 target mRNAs.

### RGLS4326 suppresses the growth of primary human ADPKD cysts

**Fig. 3:**
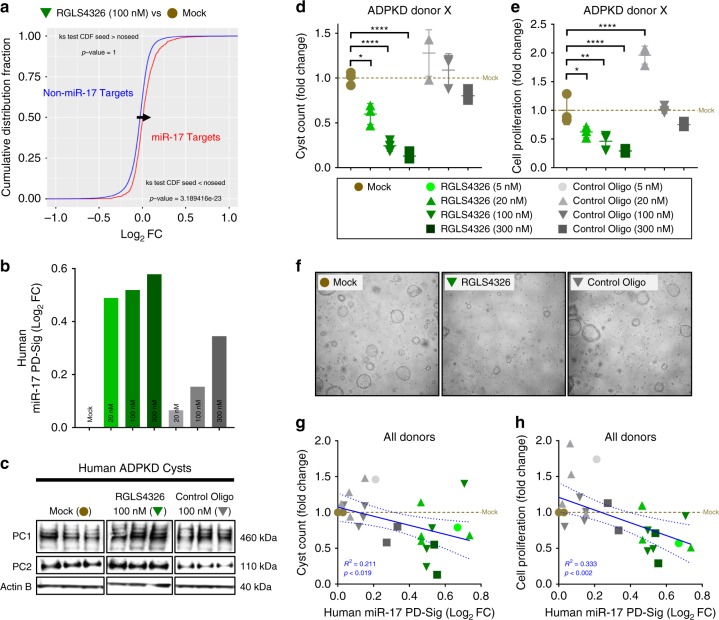
RGLS4326 suppresses the growth of primary human ADPKD cysts in vitro. **a** Primary cyst cultures derived from human ADPKD donors were transfected with RGLS4326 at 100 nM for 24 h and harvested for RNA-seq analysis. Kolmogorov-Smironov test statistics comparing the cumulative distribution of global mRNA changes between RGLS4326-treated (inverted green triangle) vs. mock-treated (brown circle) samples indicated significant de-repression of predicted miR-17 target genes (as defined by TargetScanHuman v7.1) after RGLS4326 treatment. **b** For each subsequent experiment, functional inhibition of miR-17 was assessed by measuring de-repression of human miR-17 PD-Sig^27^ in representative cyst samples (*n* = 1/treatment/dose) after 24 h transfection with RGLS4326 (shades of green) or control oligo (shades of grey) and prior to further culturing in 3D Matrigel for an additional 8 days. **c** Western blot analysis demonstrating increased expression of polycystin-1 (PC1) and polycystin-2 (PC2) 72 h following RGLS4326 treatment (*n* = 3). **d**–**e** Quantification and (**f**) representative images of 3D cyst formation showing a reduction in cyst count and proliferation 9 days following initial RGLS4326 treatment (*n* = 3). **g**–**h** Inhibition of miR-17 as measured by miR-17 PD-Sig correlates with anti-cyst and anti-proliferation activity (*n* = 4 independent experiments among 2 donors). R^2^, *p*-values and 95% confidence interval limits (dotted lines) from corresponding linear regression models were shown. Error bars represent standard deviations. *p < 0.05, **p < 0.01, ****p < 0.0001. One-way ANOVA, Dunnette’s multiple comparison test. Source data for Fig. 3**b**–**e**, and **g**–**h** is provided in Source data files

We next examined the therapeutic efficacy of RGLS4326 in vitro. To demonstrate human translational potential, we utilized primary cyst cultures derived from human ADPKD donors. We first demonstrated that RGLS4326 treatment globally de-repressed mRNAs of predicted miR-17 target genes (Fig. 3a), as well as the previously defined human miR-17 PD-Sig^27^ (Fig. 3b) in human primary ADPKD cysts. Moreover, expression of the direct miR-17 target encoded proteins polycystin-1 (PC1) and polycystin-2 (PC2) increased by ~2-fold and 4-fold, respectively, after RGLS4326 treatment (Fig. 3c). Next, we measured its effect on 3D-cyst growth and proliferation. Culturing of RGLS4326-treated primary cysts in 3D Matrigel showed a significant reduction of in vitro cyst growth and proliferation in a concentration-dependent manner (Fig. 3d–f). While the expected low magnitude of non-specific and idiosyncratic changes in PD-Sig and cyst growth were observed following control oligonucleotide treatment, our data from two individual human ADPKD donor cyst cultures demonstrate a positive correlation between miR-17 inhibition and cyst growth reduction across all treatments (Fig. 3g, h). Moreover, this effect is specific to ADPKD cells, as we observed no cytotoxicity in RGLS4326-treated non-ADPKD kidney cells where miR-17 inhibition was evident (Supplementary Fig. 3).

### RGLS4326 preferentially distributes to kidney tubules and cysts

**Fig. 4:**
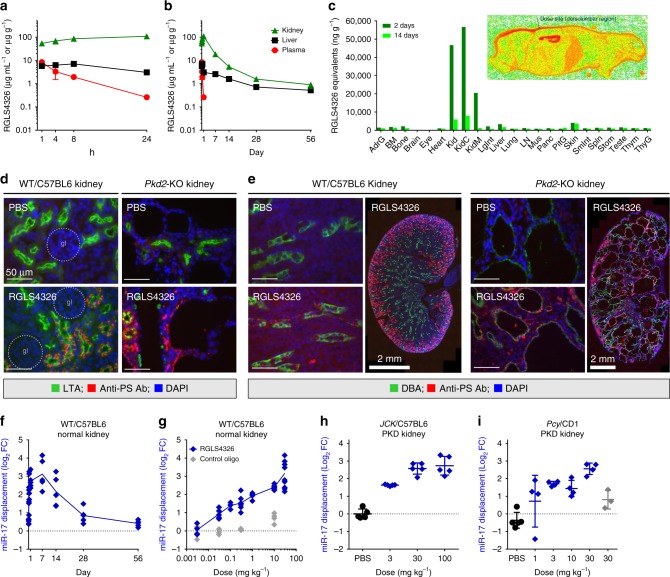
Pharmacokinetic, tissue distribution and pharmacodynamic profile of RGLS4326. **a**–**b** Kidney (green triangle), liver (black square) and plasma (red circle) exposures-vs.-time profiles of RGLS4326 following a single 30 mg kg^−1^ SC dose in WT/C57BL6 mice (*n* = 5/group). **c** Tissue distribution profile of RGLS4326 showing preferential kidney distribution based on quantitative whole-body autoradiography of [^35^S]-RGLS4326-derived radioactivity in male WT/CD1 mice 2 days (dark green) and 14 days (light green) after a single SC dose of RGLS4326 at 30 mg kg^−1^ and target radioactivity of 100 μCi kg^−1^ (*n* = 1/timepoint). Representative whole-body autoradioluminogram showing tissue distribution of radioactivity at Day 2 is shown. Red indicates intensity of radioactivity detected. **d**–**e** WT/C57BL6 (*n* = 3) or *Pkd2*-KO mice (*n* = 3) were dosed SC with PBS or 20 mg kg^−1^ of RGLS4326 on postnatal day (P)21, P22 and P23, and kidneys were harvested on P26. Kidney sections were co-stained with LTA (proximal tubules marker) or DBA (collecting ducts marker), anti-PS antibody (antibody labels RGLS4326) and DAPI. Representative merged immunofluorescence images of stained kidney sections demonstrating delivery of RGLS4326 to (**d**) proximal tubules and (**e**) collect duct cyst cells are shown. No glomerulus localization of RGLS4326 was observed in all mice tested. **f** Kidney target engagement-vs.-time profile of RGLS4326 showed kidney target engagement (blue diamond) peaked at 7 days, and continued through to at least 14 days, after a single 30 mg kg^−1^ SC dose in WT/C57BL6 mice (*n* = 5/group). **g** Dose-responsive target engagement of miR-17 in kidney tissues 7 days following a single SC dose of RGLS4326 at 0.003 (*n* = 4), 0.03 (*n* = 8), 0.1 (*n* = 4), 0.3 (*n* = 8), 3 (*n* = 4), 10 (*n* = 8), and 30 mg kg^−1^ (*n* = 10) in WT/C57BL6 mice. **h**–**i** RGLS4326 treatment dose-responsively engaged miR-17 in polycystic kidneys of two PKD mouse models 7 days after a single SC dose compare to PBS (closed black circle). RGLS4326 was dosed at 3, 30, and 100 mg kg^−1^ in *JCK*/C57BL6 mice (*n* = 5), and at 1, 3, 10, 30 mg kg^−1^ in *Pcy*/CD1 mice (*n* = 4). Control oligo (grey) was used as a negative control. Error bars represent standard deviations. Glomerulus, *gl* (dotted circle). Scale bars, 50 μm (except as specified in **e**, 2 mm). Source data for Fig. 4**a**–**c** and **f**–**i** is provided in Source data files

We next evaluated the in vivo pharmacokinetic and biodistribution profile of RGLS4326 in wild-type mice following a single 30 mg kg^−1^ SC injection. RGLS4326 is rapidly absorbed into plasma, showing T_max_ of ≤1 h, C_max_ of 8.5 µg mL^−1^, and half-life of <4 h (Fig. 4a). The rapid plasma clearance of RGLS4326 reflects the extensive distribution to tissues suchs as kidney and liver, that is commonly observed for chemically modified oligonucleotides^29^. Uniquely, RGLS4326 showed preferential kidney distribution, with kidney-to-liver (K/L) ratio of approximately 13 and 8 by C_max_ and AUC_last_, respectively (Fig. 4b). Data from a quantitative whole-body autoradiography (QWBA) study further demonstrated that RGLS4326 primarily distributes to kidney over any other organs after a single SC dose (Fig. 4c), with the majority of the administered dose eliminated in the urine. Within the kidney, chemically modified oligonucleotides are known to localize principally to proximal tubules by basolateral uptake and tubular reabsorption, whereas localization in glomeruli is minimal^11,30^. Indeed, following subcutaneous administration in wild-type mice, RGLS4326 was present mostly in proximal tubules (Fig. 4d), with lower amounts detected in cortical collecting ducts (Fig. 4e), and no glomerular uptake was noted. Importantly, we observed extensive RGLS4326 uptake in both proximal tubules and collecting duct cysts in cystic kidneys of the aggressive Pkhd1/cre;*Pkd2*^F/F^ (*Pkd2*-KO) mouse model of ADPKD following SC administration, likely linked to the severely disrupted kidney architecture in this mouse model (Fig. 4e). Additional representative immunofluorescence images of stained kidney sections are shown in Supplementary Figs. 4 and 5.

We next studied the ability of RGLS4326 to inhibit endogenous miR-17 in kidneys. Consistent with our observations of rapid kidney distribution, RGLS4326 started displacing miR-17 from HWM polysomes within 24 h after a single SC administration, with peak displacement occurring on day 7 and continuing for at least 14 days (Fig. 4f). To further examine the in vivo pharmacodynamic profile, various doses of RGLS4326 were subcutaneously administered to normal and PKD mice (*JCK*/C57BL6 and *Pcy*/CD1). RGLS4326 treatment dose-responsively displaced kidney miR-17 from HMW polysomes in both normal and PKD mice (Fig. 4g–i). In contrast, treatment with PBS or control oligonucleotides had no effect. Our analysis showed that 7-days after a single 30 mg kg^−1^ SC injection, RGLS4326 potently displaced kidney miR-17 with observed miPSA scores of 3.16, 2.58, and 2.56, representing calculated percent inhibition of 89, 83, and 83% in normal, *JCK*/C57BL6 and *Pcy*/CD1 mice, respectively.

### RGLS4326 confers efficacy in multiple PKD mouse models

**Fig. 5:**
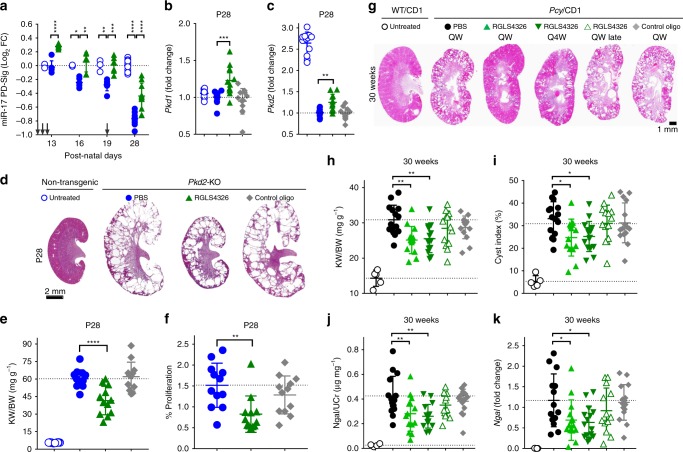
RGLS4326 confers efficacy in *Pkd2*-KO and *Pcy*/CD1 mouse models. **a**–**f** *Pkd2-*KO mice were dosed SC with PBS (closed blue circle) or 20 mg kg^−1^ of RGLS4326 (green triangle) or control oligo (grey diamond) at P10, P11, P12, and P19. Kidneys were harvested on P13, P16, P19 (*n* = 6/group), and P28 (*n* = 12), and total RNA was extracted. Age-matched untreated non-transgenic (UNT) control mice were also included for analysis (open blue circle; *n* = 3 for P13, P16, and P19 and *n* = 12 for P28). **a** *Pkd2-*KO kidneys show low level of miR-17 PD-sig, indicative of higher baseline miR-17 functional activity compared to UNT. RGLS4326 treatment de-repressed miR-17 PD-Sig. Arrows indicate dosing days. **b**–**c** RGLS4326 treatment results in de-expression of *Pkd1* and *Pkd2*. **d** Representative H&E staining of kidney sections from each treatment groups at the end of the study on P28. **e** Kidney-weight-to-body-weight ratio (KW/BW), as well as (**f**) number of proliferating cyst epithelial cells (as stained by anti-pHH3 antibody) were reduced after RGLS4326 treatment. **g**–**k** Five-weeks-old male *Pcy*/CD1 mice were dosed SC once-weekly (QW) with PBS (closed black circle) or 25 mg kg^−1^ of RGLS4326 (green triangle) or control oligos (grey diamond), or once-every-4-weeks (Q4W) with 25 mg kg^−1^ of RGLS4326 (inverted green triangle). An additional group of *Pcy*/CD1 mice were dosed SC once-weekly with 25 mg kg^−1^ of RGLS4326 starting at 15-weeks of age (QW late; open green triangle). All *Pcy*/CD1 mice (*n* = 15/group) were euthanized at 30-weeks of age, and kidney and urine samples were harvested. **g** Representative H&E staining of kidney sections from each treatment groups at the end of the study are shown. **h** KW/BW ratio, (**i**) cyst index, (**j**) urine Ngal protein, and (**k**) kidney *Ngal* mRNA expression were significantly reduced in *Pcy*/CD1 mice following RGLS4326 treatment. Corresponding values from wild-type controls (*n* = 5) are shown for reference (open black circle). Error bars represent standard deviations. *p < 0.05, **p < 0.01, ***p < 0.001, ****p < 0.0001. One-way ANOVA, Dunnette’s multiple comparison test. Scale bars, 2 mm (**d**) and 1 mm (**g**). Source data for Fig. 5**a**–**c**, **e**, **f**, and **h**–**k** is provided in Source data files

The rapidly progressing *Pkd2-*KO mouse model of ADPKD is responsive to miR-17 inhibition, where genetic knockdown of miR-17~92 attenuates disease progression^17^. Therefore, we sought to determine whether RGLS4326 treatment produces similar beneficial effects in a randomized, blinded and statistically powered efficacy study in *Pkd2-*KO mice. *Pkd2*-KO mice were dosed SC with PBS, 20 mg kg^−1^ of RGLS4326 or control oligo at post-natal day (P)10, P11, P12, and P19; and kidneys were harvested on P13, P16, P19, and P28. Compared to non-cystic control kidneys, polycystic kidneys of PBS-treated *Pkd2-*KO mice exhibit an age-dependent progressive decline in miR-17 PD-Sig, indicative of increasing miR-17 activity with disease progression (Fig. 5a). Administration of RGLS4326 reversed this decline in miR-17 PD-Sig, indicating a sustained functional inhibition of miR-17. Like previous in vitro studies, in vivo administration of RGLS4326 also upregulated the expression of the direct miR-17 target genes *Pkd1* and *Pkd2* (Fig. 5b, c). Most importantly, RGLS4326 treatment led to reduction in kidney-to-body-weight ratio (KW/BW) and decrease in cyst epithelial cell proliferation (Fig. 5d–f). In contrast, no effect was observed with the control oligonucleotide.

RGLS4326 was then evaluated in three independent long-term studies. First, we performed a randomized and statistically powered efficacy study in the slowly progressing *Pcy*/CD1 mouse model (Fig. 5g). Five-weeks-old *Pcy*/CD1 mice were treated with RGLS4326 at 25 mg kg^−1^ for 25-weeks. Reduction of KW/BW, cyst index, urine Ngal protein and kidney *Ngal* mRNA expression were observed after either weekly (QW) or monthly (Q4W) SC dosing of RGLS4326 (Fig. 5h–k). Treatment with control oligonucleotides had no effect. Initiating therapy in 15-weeks-old *Pcy*/CD1 mice (QW late) did not attenuate cyst growth, exemplifying the importance of early treatment initiation in this model.

**Fig. 6:**
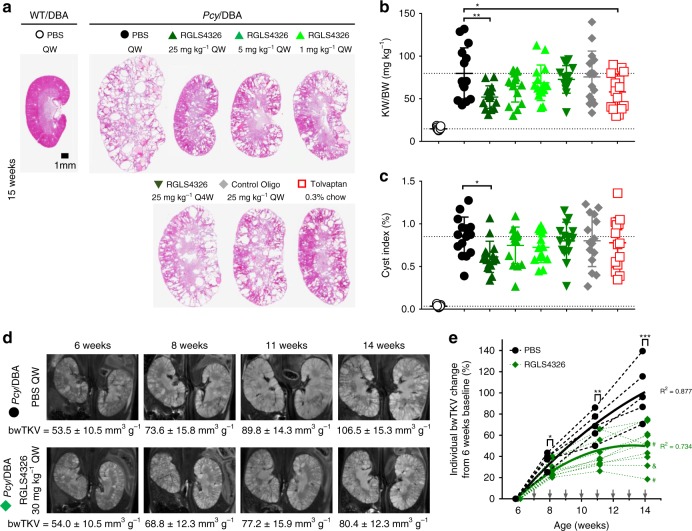
RGLS4326 confers efficacy in *Pcy*/DBA mouse model. **a**–**c** Six-weeks-old *Pcy*/DBA mice were dosed SC QW with PBS (closed black circle), or 25 mg kg^−1^ (dark green triangle), 5 mg kg^−1^ (green triangle) or 1 mg kg^−1^ of RGLS4326 (light green triangle), or Q4W with 25 mg kg^−1^ of RGLS4326 (inverted green triangle), or QW with 25 mg kg^−1^ of control oligos (grey diamond). Another group of 6-weeks-old *Pcy*/DBA mice was treated with 0.3% tolvaptan via diet ad libitum (open red square). All *Pcy*/DBA mice (*n* = 15/group) were euthanized at 15-weeks of age, and kidneys were harvested. **a** Representative H&E staining of kidney sections from each treatment groups are shown. **b** KW/BW ratio and (**c**) cyst index were significantly reduced in *Pcy*/DBA mice following weekly RGLS4326 treatment. Tolvaptan conferred efficacy based on KW/BW ratio, but not cyst index. Corresponding values from PBS-treated WT/DBA mice are shown for reference (open black circle, *n* = 5). **d** Baseline body-weight-adjusted total kidney volume (bwTKV) were obtained from 6-weeks-old male *Pcy*/DBA mice by T2-weighted MRI and used for treatment group randomization. Assigned mice were dosed SC QW with PBS (*n* = 5; closed black circle), or 30 mg kg^−1^ RGLS4326 (*n* = 10; green diamond). Representative MRI images and mean bwTKV ± standard deviations from 6-, 8-, 11-, and 14-week-old mice from each treatment groups are shown. **e** Percentage change of individual bwTKV changes from 6 weeks baseline values for each timepoints are shown. The bwTKV-vs.-time profiles for each treatment groups were fitted with second-order polynomial regression for illustration purposes. R^2^ values are shown. Arrows indicate dosing days. *Pcy*/DBA mice with stabilized and reduced bwTKV from last measurements at the end of the study are indicated by & and #, respectively. Error bars represent standard deviations. *p < 0.05, **p < 0.01, ***p < 0.001. One-way ANOVA, Dunnette’s multiple comparison test. Scale bar, 1 mm. Source data for Fig. 6**b**, **c** and **e** is provided in Source data files

In a second long-term study, we examined RGLS4326 in the more aggressive *Pcy*/DBA mouse model^31^. Six-weeks-old *Pcy*/DBA mice were treated with QW doses of RGLS4326 at 25, 5, or 1 mg kg^−1^ for 9-weeks. Tolvaptan is currently the only approved treatment for ADPKD. Therefore, we also performed a head-to-head comparison between RGLS4326 and tolvaptan in the *Pcy*/DBA model. RGLS4326 demonstrated a dose-dependent reduction in KW/BW and cyst index following 9 consecutive weeks of QW SC injections (Fig. 6a–c). However, monthly RGLS4326 treatment did not confer efficacy in the *Pcy*/DBA model. Tolvaptan treatment reduced KW/BW but did not improve cyst index in this head-to-head comparison.

Finally, in the third long-term study, we prospectively monitored disease progression in *Pcy*/DBA mice treated with either PBS or 30 mg kg^−1^ RGLS4326 SC QW. Disease progression in ADPKD patients can be tracked by height-adjusted total kidney volume (htTKV) using magnetic resonance imaging (MRI)^32,33^. Hence, we monitored *Pcy*/DBA mice using the comparable MRI-based body-weight-adjusted TKV (bwTKV) (Fig. 6d). RGLS4326 treatment was initiated in 7-week-old *Pcy*/DBA mice in which substantial cysts had already formed. In an 8-week efficacy study, bwTKV increased almost linearly in PBS-treated *Pcy*/DBA mice, whereas growth of polycystic kidneys started to plateau after only 4 weeks of RGLS4326 treatment (Fig. 6e). One out of ten *Pcy*/DBA mice treated with RGLS4326 showed stabilization and two out of ten mice showed a reduction of bwTKV.

### RGLS4326 improves dysregulated gene network expression in PKD

**Fig. 7:**
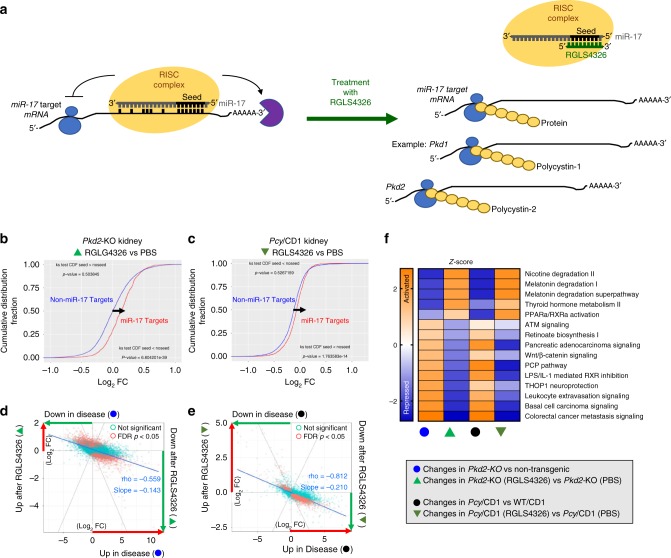
RGLS4326 improves expression of dysregulated gene networks in PKD models. **a** Schematic illustration of RGLS4326-mediated inhibition of miR-17. RGLS4326 displaces miR-17 from the translationally active polysome fractions and de-represses miR-17 target genes including *Pkd1* and *Pkd2* and their encoded proteins PC1 and PC2. **b**–**e** RNA-seq analysis was performed to compare mRNA expression profiles between kidneys from non-transgenic (*n* = 12), PBS-treated *Pkd2*-KO (*n* = 8), RGLS4326-treated *Pkd2*-KO (*n* = 11), and control oligo-treated *Pkd2*-KO mice (*n* = 8). RNA-seq analysis was also performed from wild-type (*n* = 2), PBS-treated *Pcy*/CD1 (*n* = 4), RGLS4326-treated *Pcy*/CD1 (*n* = 4), and control oligo-treated *Pcy*/CD1 mice (*n* = 3). **b**–**c** Kolmogorov-Smironov test statistics comparing the cumulative distribution of global mRNA changes between RGLS4326-treated vs. PBS-treated kidney samples indicated significant de-repression of predicted miR-17 target genes (as defined by TargetScanMouse v7.1) after RGLS4326 treatment in *Pkd2*-KO (green triangle) and *Pcy*/CD1 model (inverted green triangle). **d**–**e** Comparative differential expression analysis demonstrated a clear trend in global transcriptomic changes where dysregulated gene expression in *Pkd2*-KO (blue circles) and *Pcy*/CD1 kidneys (black circles) (*x*-axis) were improved after RGLS4326 treatment (*y*-axis). Rho-values and slopes from corresponding Spearman’s correlations are shown. RGLS4326 treatment improves the expression of 994 genes in *Pkd2-*KO (*n* = 11) and 658 genes in *Pcy*/CD1 kidney (*n* = 4) (FDR < 0.05 and Log_2_FC > ∣0.5∣). **f** Top 15 pathways as predicted by the ingenuity pathway analysis software (based on ∣*z*-scores∣) potentially responsible for the gene changes are shown. Positive *z*-scores (shades of orange) indicate activation, while negative z-scores (shades of blue) indicate repression. Source data for Fig. 7**f** is provided in Source data files

We have shown that RGLS4326 displaces miR-17 from the translationally active HMW polysomes, de-represses miR-17 target genes including *Pkd1* and *Pkd2* and their encoded proteins PC1 and PC2, and is efficacious in PKD models (Fig. 7a). To gain further insights into RGLS4326 mechanism-of-action (MOA) in the context of PKD treatment, we began by comparing the kidney global mRNA expression profiles in the *Pkd2*-KO and *Pcy*/CD1 models following RGLS4326 treatment vs. their respective PBS-treated controls. We found that RGLS4326 treatment had a significant transcriptome-wide impact and globally de-repressed mRNAs of predicted miR-17 target genes in cystic kidneys of both PKD models (Fig. 7b, c). In parallel, we also compared kidney expression profiles of the two PKD mouse vs. their respective non-diseased strain-matched controls. As expected, numerous genes were aberrantly expressed in cystic kidneys of these mice compared to their non-diseased counterparts (Fig. 7d, e; *x*-axis). Comparative differential expression analysis showed a clear trend in global transcriptomic profiles where the expressions of dysregulated genes in the *Pkd2*-KO and *Pcy*/CD1 cystic kidneys were improved after RGLS4326 treatment (rho = −0.559 and −0.812, respectively) (Fig. 7d, e; *y*-axis). Specifically, RGLS4326 treatment improved the expression of a total of 994 genes in *Pkd2*-KO kidneys and 658 genes in *Pcy*/CD1 kidneys (FDR < 0.05 and Log_2_FC > |0.5|). We next used ingenuity pathway analysis software to identify gene networks and pathways that were improved in the PKD models following RGLS4326 treatment. The top 15 pathways potentially responsible for the gene changes are shown in Fig. 7f. Consistent with our previous observations in miR-17~92 genetic deletion studies and the known oncogenic role of miR-17, RGLS4326 treatment was associated with normalization of metabolism pathways^34^ (e.g., PPARα/RXRα) and inhibition of pro-proliferative oncogenic pathways^21^ (e.g., Wnt pathway).

### Preclinical safety assessment of RGLS4326

Oligonucleotides containing 2′-deoxy-2′-fluoro nucleosides have the potential to inhibit DNA repair and to induce mitochondrial toxicity^35–37^. We therefore assessed the genotoxic and mitochondrial toxicity potential of RGLS4326, which contains three 2′-deoxy-2′-fluoro nucleosides. No genotoxicity was observed in bacterial mutagenicity assay, in vitro human lymphocyte micronucleus assay, or an in vivo micronucleus and comet assay in mice following three daily SC doses up to 2000 mg kg^−1^ (Supplementary Table 2). In addition, no mitochondrial toxicity was observed in vitro in human cells under conditions that require mitochondrial oxidative metabolism, and in vivo where mitochondrial density and morphology were examined by transmission electron microscopy in liver and mitrochondria-rich cardiac tissues following repeat SC dosing of RGLS4326 in monkeys.

Given that certain oligonucleotides can evoke acute inflammatory response, we also evaluated the potential pro-inflammatory properties of RGLS4326 in rat tissue slice assays ex vivo and acute mouse studies in vivo. RGLS4326 was found to have low risk for pro-inflammatory effects, as no changes were observed in the well-characterized proinflammatory genes *Oas1a* and *Ifit3* following RGLS4326 treatment.

**Fig. 8:**
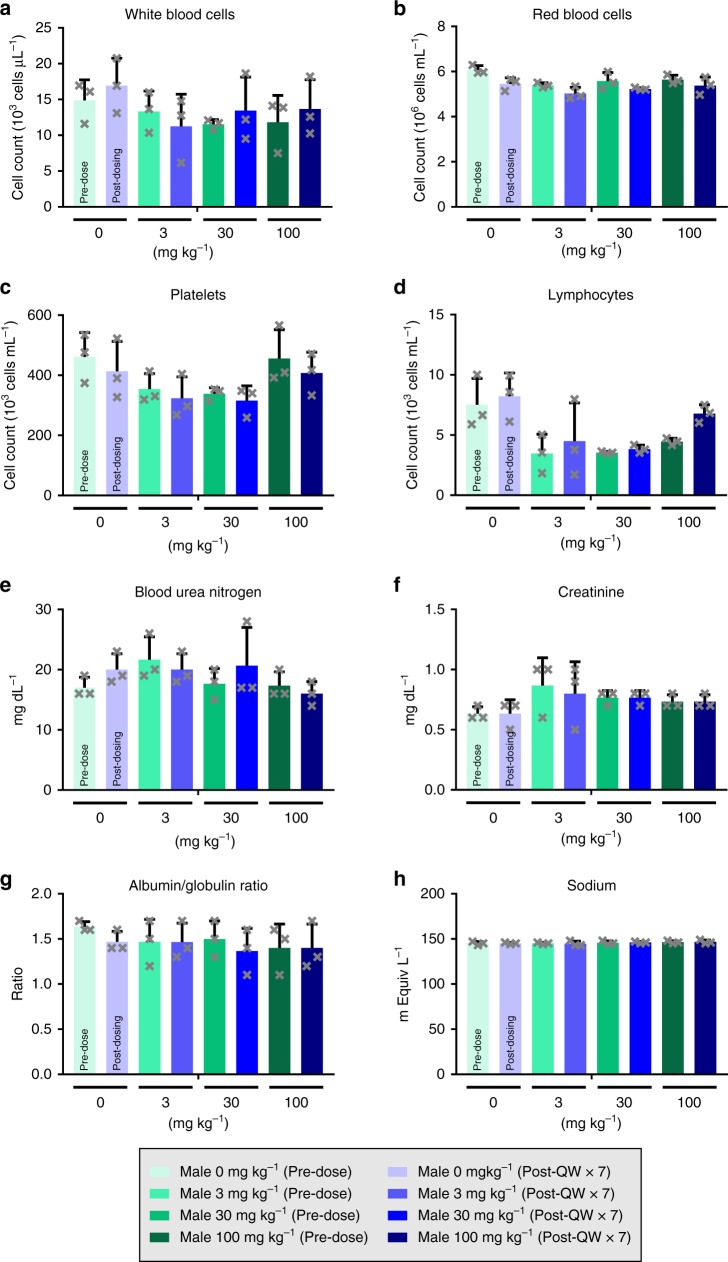
RGLS4326 does not cause hematopoietic or kidney toxicity in monkeys. Male cynomolgus monkeys (*n* = 3/group) were dosed SC QW with 0, 3, 30, or 100 mg kg^−1^ of RGLS4326 for 7 consecutive weeks (QWx7). Prior to the study, animals were acclimated to the study room for 14 days. On the final day of acclimation, animals weighed between 2.8 and 6.1 kg and were 3 to 6 years of age. Blood was collected for hematology and serum chemistry analysis before and at 48 h after the last dose. Specimens were analyzed using an Advia 120 automated hematology analyzer and an AU680 chemistry analyzer. Comparison between pre-dose and post-QWx7 values for each treatment groups indicated no hematological abnormalities (**a**–**d**) and no renal toxicity (**e**–**h**) following RGLS4326 treatment. Error bars represent standard deviations. Source data for Fig. 8**a**–**h** is provided in Source data files

Genetic deletion of miR-17~92 in adult mice has been shown to reduce various hematopoietic cell populations^25^. We therefore examined the effect of RGLS4326 on these cell populations in cynomolgus monkeys and CD1 mice after repeat RGLS4326 SC administrations. Consistent with the preferential kidney distribution profile of RGLS4326, repeat dosing of RGLS4326 in monkeys or mice even at supratherapeutic ranges did not affect the various cell populations, including white blood cells, red blood cells, platelets and lymphocytes (Fig. 8a–d and Supplementary Fig. 6A–D). More importantly, although renal toxicity has been observed with certain classes of oligonucleotide therapeutics, RGLS4326 treatment had no detrimental effect on kidney function in either species (Fig. 8e–h and Supplementary Fig. 6E–H). Altogether, our data shows that RGLS4326 has a favorable preclinical safety profile.

## Discussion

Chronic kidney diseases are of significant societal burden on par with cancer and cardiovascular diseases; however, drug development in this area, including ADPKD, has been limited. Here, we report the discovery and preclinical evaluation of RGLS4326 as a potential disease-modifying treatment for ADPKD.

Several key attributes make RGLS4326 an attractive potential therapeutic agent for ADPKD. First and foremost, RGLS4326 is efficacious in multiple PKD mouse models across a range of doses and treatment regimens. The magnitude of effectiveness as determined by MRI-based TKV (50% reduction in bwTKV) is comparable to that observed for tolvaptan treatment in the TEMPO 3:4 clinical trial (49% improvement in yearly TKV)^33^. Considering that RGLS4326 and tolvaptan function through independent mechanisms, combining these two distinct drug classes may produce synergistic beneficial effects and additional investigations are warranted.

Secondly, we provide several lines of evidence that RGLS4326 preferentially distributes to kidney and renal tubules, thereby minimizing exposure and potential toxicity to other cells and tissues where miR-17 might also play important roles, such as hematopoietic cells. While the mechanisms for the differential exposure are not entirely clear, our data showed that RGLS4326 was >8-fold more abundant in kidney compared to liver following SC administration, perhaps due to its short 9-nt length compared to other oligonucleotide therapeutics. The QWBA data also showed that RGLS4326 has very low exposure in other tissues such as adrenal gland, spleen, bone marrow, and lymph nodes. Indeed, we observed no hematological abnormalities after RGLS4326 treatment in both mice and monkeys, even at very high supratherapeutic doses.

Thirdly, RGLS4326 remains active in the kidney for at least 14 days. In contrast, tolvaptan, as well as other medications that are currently in clinical trials for ADPKD, are administered once or twice daily. Indeed, we have demonstrated that RGLS4326 treatment, ranging from weekly and up to monthly administrations, confers efficacy in different mouse models of PKD. The longer half-life in kidney, which allows for less frequent dosing, is a desirable feature for chronic diseases like ADPKD that require long-term treatment.

miRNA-based drugs have a unique MOA compared to traditional drugs. miRNAs simultaneously repress many mRNA targets, thereby affecting multiple signaling pathways. Moreover, miRNAs function as rheostats rather than on-off switches. Whereas traditional drugs often inhibit (or activate) a single or few pathways, a miRNA-based therapeutic can comprehensively modulate multiple pathways. Consistent with this idea, we showed that RGLS4326 treatment improves the expression of many pathogenic gene networks and pathways in PKD. Importantly, this coordinated rewiring of the polycystic kidney transcriptome towards a normal kidney profile exemplifies the potential of RGLS4326 as a disease-modifying treatment for ADPKD. Finally, one of the most intriguing features of RGLS4326 is its potential direct mechanistic link to ADPKD. We and others have shown that miR-17 binds to the 3′UTRs of *PKD1* and *PKD2* and inhibits their expression^18,38,39^. Accordingly, RGLS4326 promotes the expression of both genes in murine and human kidney cells. It is therefore tempting to speculate that part of the RGLS4326 therapeutic efficacy is via direct *PKD1/2* de-repression. This MOA is especially of relevance in the context of hypomorphic mutations, where RGLS4326-mediated increase in *PKD1/2* gene dosage above a critical threshold may alleviate ADPKD^2^.

In conclusion, we have discovered a first-in-class anti-miR-17 oligonucleotide RGLS4326 that has promising potential as drug treatment for ADPKD. RGLS4326 has favorable potency, stability, safety, pharmacokinetic, and pharmacodynamic characteristics, including preferential distribution to kidney. RGLS4326 attenuates cyst growth in human ADPKD models in vitro and is efficacious in multiple PKD mouse models in vivo. Our data strongly support the clinical development of RGLS4326 for ADPKD.

## Methods

### Cell lines

Mouse IMCD3 kidney collecting duct cells (CRL-2123; ATCC) were cultured with 1:1 DMEM/F-12 medium (ATCC) supplemented with 10% FBS and 1% penicillin-streptomycin. Mouse M1 kidney collecting duct cells (ATCC CRL-2038) were cultured with 1:1 DMEM/F12 supplemented with 2.5 mM L-glutamine, 15 mM HEPES, 0.5 mM sodium pyruvate, 1.2 g L^−1^ sodium bicarbonate, 0.005 mM dexamethasone, and 5% FBS. Mouse DBA-WT and DBA-PKD kidney collecting duct cells and mouse LTL-WT and LTL-PKD kidney proximal tubular cells (Discovery BioMed, Inc) were cultured with 1:1 RenaLife Complete Medium (Lifeline Cell Technology) and Advance MEM medium (Invitrogen) supplemented with 5% FBS, 4 mM L-Glutamine and 1% of penicillin-streptomycin^40^. Mouse Myc-driven HCC cell line obtained from Dr. Dean Felsher Lab were cultured in DMEM (Invitrogen) supplemented with 10% FBS^41^. Mouse MDCT kidney distal convoluted tubule cells (ATCC CRL-3250) were cultured in 1:1 DMEM/F-12 supplemented with 5% FBS, 1 g L^−1^ glucose, and 1 mM sodium pyruvate. Mouse NIH/3T3 (ATCC CRL-1658), Hep-C7 (ATCC CRL-2026), and HeLa (ATCC CCL-2) cells were cultured according to ATCC’s protocols. All cells were incubated in an atmosphere of 95% air and 5% CO_2_ at 37 °C.

### Ex-vivo tissue slice assay

Two hundred micro (μm) thin precision-cut tissue slices were freshly prepared from kidneys of 8-week-old WT/C57BL6 mice, or kidneys and livers from 8-week-old Sprague-Dawley rats using Brendel/Vitron tissue slicer under cold oxygenated preservation solution V7 (Vitron) and incubated in Waymouth’s medium (Gibco) supplemented with 10% FBS and 1% Antimycotic at 37 °C, 95% O_2_, 5% CO_2_ on a rotating Vitron incubator. Two hours post-slicing, the medium was replaced with fresh Waymouth’s medium containing RGLS4326, oligo control or PBS, and replenished every 24 h. For pro-inflammatory liability assessment, rat liver and kidney slices were incubated with 5 μM RGLS4326 for 24 h and 48 h, respectively, prior to RNA extraction and analysis. For miR-17 PD-Sig analysis, mouse kidney slices were incubated at 10 μM RGLS4326 for 72 h.

### Primary human ADPKD cyst assay

Primary human ADPKD cyst cells were obtained from PKD Research Biomarker and Biomaterial Core at the University of Kansus Medical Center (KUMC). Informed consent was obtained from all the human participants and the protocol for the use of surgically discarded kidney tissues complied with federal regulations and was approved by the Institutional Review Board at the University of Kansas Medical Center. Cells were cultured in DMEM/F12++(cat # 10565–018, Gibco) supplemented with 5% FBS, 5 μg kg^−1^ insulin, 5 μg mL^−1^ transferrin and 5 ng mL^−1^ sodium selenite and incubated in an atmosphere of 95% air and 5% CO2 at 37 °C until 80% confluency. Tryptinized cells (100,000 cells/well in a 6 well plate) were reverse transfected with Lipofectamine RNAiMAX reagent (cat # 13778–150, Life Technologies) with RGLS4326, oligo control or PBS. Twenty four hours later, cells were tryptinized, counted and plated at 4000 cells/well density in 130 μl of media plus Matrigel (cat # 354234, Corning) in a 96-well plate (cat # 353072, Corning). The gel was allowed to solidify before 130 μl of transfection media (117 μl of complete media and 13 μl of transfection complex) was added to the top of the matrigel. Media was replenished every 72 h until 8 days post plating when the cyst number and volume were assessed. Images (twenty-eight 24-bit colour TIFF images at 2448-by-1920 pixels at 72 dpi) from each well were captured with an Olympus DP26 camera (Olympus Corporation) and were comprised of 28 images “stacks” taken 150 microns apart. Image processing took place using a custom script written in the R language employing the EBImage Bioconductor image processing library^42^. Images from one well were processed as a batch, with each image from each image stack undergoing segmentation analysis to detect objects in each image of a different contrast. Per detected object, the following statistics were collected: mean radius in pixels, CV of the radius, and the eccentricity (i.e., deviation from circularity where a perfect circle = 0). Images were filtered to remove noise and non-cyst objects by removing objects less than 15 and greater than 200 pixel mean radius, greater than 0.2 CV radius and greater than 0.75 eccentricity. Images were considered in the context of the entire well because larger cysts span more than one image in the image stack. Cyst objects detected in the same location in sequential images were considered to be the same cyst. Assuming the cyst was spherical in shape, the largest cyst object in the series of objects was chosen, its radius (r) was found and the volume of the cyst was calculated using the formula 4/3 πr^3^. This same exercise was performed using all wells (i.e., all image stacks) for all donors.

### Animals

*Pkd2-*KO*, JCK*/C57BL6 (C57Bl/6J-nek8^jck^, Crown Bio), *Pcy*/CD1 (CD-1-pcy^lusm^, Crown Bio), *Pcy*/DBA (DBA/2FG-pcy, Kyudo Co), 129 × 1/SvJ (Jackson laboratory), WT/C57BL6 mice (C57BL/6J, Jackson laboratory), WT/DBA (Charles River), CD1 mice (Charles River and SNBL-USA), Sprague-Dawley rats (Charles River), and Cynomolgus Monkey (SNBL-USA) were used in this study. All animal experiments were conducted in accordance with the Institutional AAALAC Guidelines and approved by the Institutional Animal Care and Use Committees at Explora, UT Southwestern, InVicro, and SNBL-USA where the experiments were performed.

### Luciferase sensor assay

HeLa cells were transfected for 4 h with Synthetic miRNA Target GoClone Reporters (harboring two fully complementary miRNA binding sequence of interest at 3′UTR of the luciferase gene; Active Motif) and Human pre-microRNA Expression Construct Lenti-miRNAs of interest (System Biosciences) using Lipofectamine LTX-Plus reagent (Life Technologies). Cells were seeded overnight prior to transfection with anti-miR for 24 h using Lipofectamine RNAiMAX (Life Technologies). Luciferase activity was measured using LightSwitch Luciferase assay kit (Active Motif). Raw luciferase data was background subtracted and normalized to untreated control. EC_50_ values were estimated using GraphPad Prism software v7.04.

### miRNA expression analysis

Reverse transcription was performed using the TaqMan® MicroRNA reverse transcription kit (Applied Biosystem) and TaqMan® miRNA assays (Applied Biosystem). Real-time PCR was performed using TaqMan® Universal PCR Master Mix II according to the manufacturer’s protocol. List of TaqMan® miRNA Assays used is shown in Supplementary Table 3.

### Gene expression analysis

Total RNA was isolated using the RNeasy mini kit (Qiagen). Reverse transcription and preamplification reactions were performed using Bio-Rad T100 Thermocycler (Bio-Rad) according to Fluidigm’s protocol. Quantitative real-time PCR was performed using the BioMark Real-Time PCR System Dynamic Array 192.24 IFC (Fluidigm) according to the manufacturer’s protocols. Data were analyzed using BioMark Real-Time PCR analysis software version 2 (Fluidigm). Relative gene expression values were determined using the ΔΔCT method. The mouse miR-17-PD-Sig was calculated as the average log2 fold change in expression of 18 mouse miR-17 target genes (*Plekha3, Rhoc, D030056l22rik, Clock, Polq, Mtf1, E2f1, Tgfbr2, P2rx4, Nagk, Mink1, Zfp367, Fyco1, Pfkp, Ddhd1, Cdc37l1, Wfs1,* and *St6galnac6*) normalized by 6 housekeeping genes (*Tbp, Gusb, Rplp0, Hprt1, B2m,* and *Pak1ip1*) compared to Mock (transfection) or PBS (free-uptake) for in vitro studies, while a representative 10 mouse genes (*Plekha3, Clock, Polq, Mtf1, Nagk, Mink1, Zfp367, Fyco1, Ddhd1, and Cdc37l1*) was used for in vivo *Pkd2*KO mouse studies. The human miR-17 PD-Sig^27^ was calculated as average log2-fold change in expression of 13 human miR-17 target genes (*AMPD3, BTG3, C7ORF43, CROT, ENPP5, LIMK1, MINK1, NAGK, NKIRAS1, PLEKHA3, PTPN4, TBC1D9*, and *TGFBR2*) normalized by 6 housekeeping genes (*B2M, GUSB, HPRT1, RPLP0, TBP*, and *UBC*). The list of probes used is shown in Supplementary Table 4.

### RNA-sequencing analysis

RNA-Seq libraries were prepared using TruSeq stranded mRNA LT sample prep kit and ran on NextSeq 500 sequencer per manufacturer’s protocol (Illumina). Sequencing quality was assessed with the FastQC tool and trimmed with TrimGalore. Samples between 2 × 10^7^ and 6 × 10^7^ paired-end and 75 bps long reads were mapped with STAR on *Mus musculus* (GCA000001635.2) genome assembly GRCm38 (mm10) from Genome Reference Consortium. Uniquely mapped reads were counted in genes using HTseq, while mapping and counting quality were assessed by Picard (http://broadinstitute.github.io/picard/) and RSeQC tool. Differential expression was performed using edgeR v3.20.2. Kolmogorov-Smironov test statistics comparing cumulative distribution fraction (CDF) of global mRNA changes for predicted miRNA targets based on TargetScanMouse v7.1 or TargetScanHuman v7.1 were assessed^43^. Ingenuity Pathways Analysis software was used to predict putative gene networks involved with input differential mRNA expression data.

### Quantification of RGLS4326 in plasma and tissues

Plasma (0–8 h post dose) and tissue concentrations of RGLS4326 were measured using high-performance liquid chromatography time-of-flight mass spectrometry (HPLC-TOF). Plasma samples from 24–1344 h post dose were quantified using high-performance liquid chromatography with fluorescence detection (HPLC-FL). For HPLC-TOF, RGLS4326 was isolated from 100 μL of plasma or 20 mg of tissue by liquid-liquid extraction followed by solid-phase extraction steps. For HPLC-FL, 30 μL plasma was subjected to proteinase digestion followed by precipitation and hybridization of the supernatant to fluorescent probe complementary to RGLS4326, prior to injection into fluorescence detector-equipped HPLC. Calibration curve samples were prepared by spiking blank plasma or tissue homogenates with known concentrations of RGLS4326 and co-extracting calibration-samples alongside with samples. Mass spectrometry or fluorescent signal from samples were extrapolated to the calibration curve to obtain concentration measurements. Signal analysis was performed using MassHunter Version 7.0 (Agilent Technologies). A value of zero was assigned to concentrations below the limit of quantification. Pharmacokinetic parameters were determined using Phenoix® WinNonlin® version 6.3 (Certara L.P. (Pharsight), St. Louis, MO).

### Western blot analysis

Cells were lyzed in PIERCE RIPA buffer (ThermoFisher Scientific). A total of 20 μg of protein were loaded on a NuPAGE 3–8% Tris-Acetate gel (Invitrogen), transferred to 0.45 μm PVDF membranes, immunoblotted with anti-PC1 (1:200, 7E12, sc-130554, SantaCruz), anti-PC2 (1:200, D-3, sc-28331, SantaCruz) or anti-ActinB (1:5000, BA3R, Invitrogen) antibodies, and detected with chemiluminescence using standard protocols. Original western blot images are shown in Supplementary Fig. 7.

### Histology, cyst index, and immunofluorescence staining

*Pkd2-*KO left kidney was perfused with cold PBS and 4% PFA prior to collection. All other mouse kidneys were collected, fixed in 10% formalin, dehydrated, and embedded in paraffin using a standard protocol. Samples were sectioned at 5 μm and subjected to hematoxylin and eosin staining. Cyst index (cystic area/total kidney section surface area) was performed using ImageJ analysis software. For immunofluorescence staining, the following antibodies and dilutions were used: anti-PS (Regulus Therapeutics Inc, 1:1000), anti-phosphohistone H3 (1:400, Sigma-Aldrich H0412), Biotinylated Dolichos biflorus agglutinin (DBA; Vector Laboratories, 1:500), and Fluorescein Lotus tetragonolobus agglutinin (LTA; Vector Laboratories, 1:200). Secondary antibodies were conjugated to Alexa Fluor 488 or Alexa Fluor 594 (Molecular Probes, 1:400).

### Quantification of urinary Ngal

Enzyme-linked immunosorbent assay (ELISA) was performed on mouse urine samples (diluted 1:200) with Mouse Lipocalin-2/NGAL Quantikine ELISA kit (R&D Systems) according to the manufacturers’ instructions. Values were normalized with urine creatinine (UCr) level and expressed as μg Ngal per mg UCr.

### Quantitative whole-body autoradiography (QWBA)

Tissue distribution was assessed based on QWBA of [^35^S]-RGLS4326-derived radioactivity in male CD-1 mice following a single subcutaneous (SC) dose of RGLS4326 at 30 mg kg^−1^ and a target radioactivity of 100 μCi kg^−1^. At approximately 48 and 672 h post dose, mice were euthanized, and carcasses were submerged in a dry ice/hexane bath. The frozen carcasses were individually set in a mold, submerged in 5% (w v^−1^) low viscosity carboxymethylcellulose (CMC), and embedded by placing the stage (mold) in a dry ice/hexane bath. Frozen blocked carcasses were removed from dry ice/hexane bath, and 1/4-inch holes were drilled into the sample blocks and filled with calibration standards containing known quantities of [^35^S]-RGLS4326, prior to sectioning. Thirty microns thick sagittal sections of the CMC-embedded mouse carcasses, including calibration standards were sectioned and imaged using phosphor imaging plates. Autoradioluminograms of the sections were compared to calibration standards to obtain a measurement of the concentration of [^35^S]-RGLS4326.

### Efficacy studies

For *Pkd2-*KO mouse model, mice were randomly assigned and administered by SC injection with phosphate-buffered saline (PBS), 20 mg kg^−1^ of RGLS4326 or oligo control on post-natal day (P)10, P11, P12, and P19. All animals were euthanized, and samples harvested at P13, P16, P19, and P28. Non-transgenic strain-matched mice were also sacrificed on same days. Each group had 5–12 mice. Investigators were blinded to treatment groups until predetermined analysis was completed. For *Pcy*/CD1 mouse model, 5-weeks old mice were randomly assigned and dosed with RGLS4326 or control oligo at 25 mg kg^−1^ or PBS by SC injections once-weekly (QW) or monthly (Q4W) for 25 consecutive weeks. An additional group of 15-weeks-old *Pcy*/CD1 mice were dosed with 25 mg kg^−1^ RGLS4326 SC QW for 15 consecutive weeks. Five untreated male WT/CD1 mice were included for comparison. All animals were euthanized, and samples harvested at 30 weeks of age (7 days after the last dose). Each group had 15 mice. For *Pcy*/DBA mouse model, 6-weeks old male mice were randomly assigned and dosed SC with PBS, control oligonucleotide at 25 mg kg^−1^, or RGLS4326 at 1, 5, or 25 mg kg^−1^ QW or with RGLS4326 at 25 mg kg^−1^ Q4W for 9 consecutive weeks. An additional group of mice were treated with 0.3% tolvaptan via diet from 6 to 14 weeks of age. Five male WT/DBA mice treated with PBS SC QW were included for comparison. All animals were euthanized, and samples harvested at 15 weeks of age (7 days after the last dose). Each group had 15 mice. For magnetic-resonance imaging (MRI) study, male *Pcy*/DBA mice were imaged at baseline at 6 weeks of age prior to treatment group assignment. All mice were dosed SC QW with PBS (*n* = 5) or 30 mg kg^−1^ RGLS4326 (*n* = 10) starting at 7 weeks of age for 8 consecutive weeks. Additional MRI was performed at 8, 11, and 14 weeks of age.

### Magnetic resonance imaging (MRI) protocol

MRI scanning was accomplished using an 11.7T Bruker Biospec/Avance III system and a 38 mm volume RF coil. After induction of anesthesia, mice were placed inside the coil with respiratory monitoring, and warm air heating was used throughout. After scout scans for slice planning, a multi-slice T2-weighted RARE protocol (repetition time = 1.2 s; RARE factor = 8; effective echo time = 22.5 ms), was used with 0.75 mm thick contiguous coronal slices used to cover the extent of both kidneys. The field of view was 30 × 30 mm over a 256 × 256 matrix.

### In vivo acute pro-inflammatory liability mouse study

Male 6–10 weeks-old 129 × 1/SvJ mice were administered a single SC dose of RGLS4326 at 300 mg kg^−1^ or PBS and euthanized at 96 h. Kidney and liver samples were harvested and snap-frozen for RNA extraction. Relative gene expression of *Oasl2* and *Ifit1* in kidney and liver were determined using the ΔΔCT method compared to PBS. Clinical chemistry was assessed by the Axcel Blood Chemistry Analyzer (Alfa Wassermann, Caldwell, NJ).

### Reporting summary

Further information on research design is available in the Nature Research Reporting Summary linked to this article.

## Supplementary information

Supplementary Information

Reporting Summary

Source Data

## Data Availability

The RNA-seq data has been deposited in the NCBI Gene Expression Omnibus repository under accession number GSE134721. The source data underlyging Figs. 1b–f, 2a–f, 3b–e, g–h, 4a–c, f–i, 5a–c, e–f, h–k, 6b–c, e, 7f, and 8a–h, as well as Supplementary Figs 1A–E, 2C–D, 3A–D, and 6A–H, are provided as a Source Data File.
