# Supplementary material for: Discovery and preclinical evaluation of anti-miR-17 oligonucleotide RGLS4326 for the treatment of polycystic kidney disease
Source: Nat Commun. 2019 Sep 12;10:4148. doi: 10.1038/s41467-019-11918-y (PMC6742637; doi:10.1038/s41467-019-11918-y)
Supplement: Supplementary file 1 — Supplementary Information [file 41467_2019_11918_MOESM1_ESM.pdf]

SUPPLEMENTARY INFORMATION:

Discovery and preclinical evaluation of anti-miR-17 oligonucleotide RGLS4326 for the treatment of polycystic kidney disease

AUTHORS:

Edmund C. Lee[1,*], Tania Valencia[1], Charles Allerson[1], Annelie Schairer[1], Andrea Flaten[2], Matanel Yheskel[2], Kara Kersjes[1], Jian Li[1], Sole Gatto[1], Mandeep Takhar[1], Steven Lockton[1], Adam Pavlicek[1], Michael Kim[1], Tiffany Chu[1], Randy Soriano[1], Scott Davis[1], John R. Androsavich[1], Salma Sarwary[1], Tate Owen[1], Julia Kaplan[1], Kai Liu[1], Graham Jang[1], Steven Neben[1], Philip Bentley[1], Timothy Wright[1], Vishal Patel[2,*]

AFFILIATIONS:

[1]Regulus Therapeutics Inc., San Diego, California.

[2]Department of Internal Medicine and Division of Nephrology, University of Texas Southwestern Medical Center, Dallas, Texas.

***TO WHOM CORRESPONDENCE SHOULD BE ADDRESSED:**

Email: elee@regulusrx.com (E.C.L); vishald.patel@utsouthwestern.edu (V.P.)

Suppplementary Figure 1

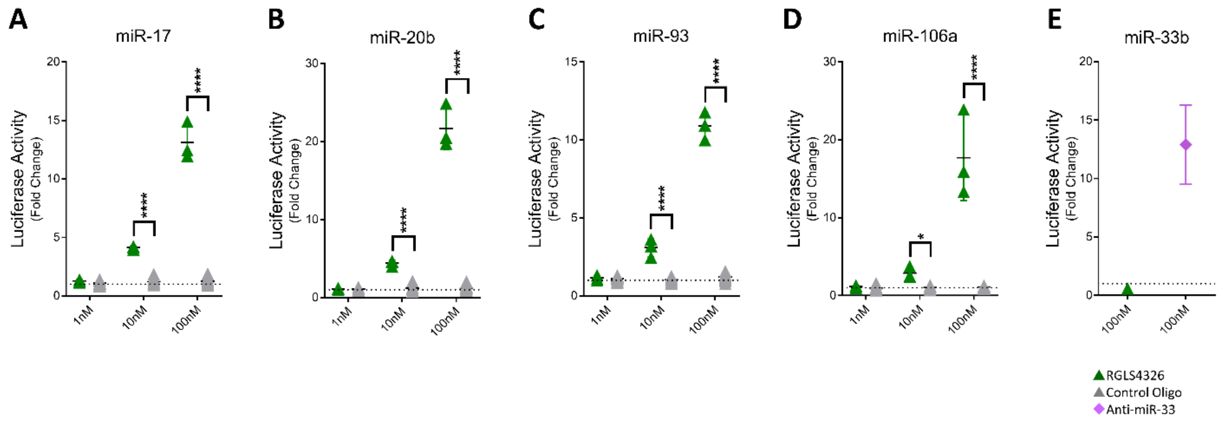

Supplementary Figure 1. ***RGLS4326 inhibits miR-17 family of miRs in luciferase sensor assays***. RGLS4326 (green triangle) dose-responsively inhibited miR-17 family members, including **(A)** miR-17, **(B)** miR-20b, **(C)** miR-93 and **(D)** miR-106a, in corresponding HeLa cell luciferase sensor assays 24 h following transfection (1-100 nM; n=3/group). Control oligo (grey triangle) containing the same chemical-modification, length, and design as RGLS4326, but different base pair sequence, was used as a negative control. **(E)** RGLS4326 has no activity against miR-33b in corresponding HeLa cell luciferase sensor assay 24 h following transfection at 100 μM (mean ± standard deviations; n=3). An anti-miR-33 oligonucleotide (magenta diamond) was used as a positive control. Error bars represent standard deviations. *p<0.05, ****p<0.0001. One-way ANOVA, Dunnette's multiple comparison test.

Suppplementary Figure 2

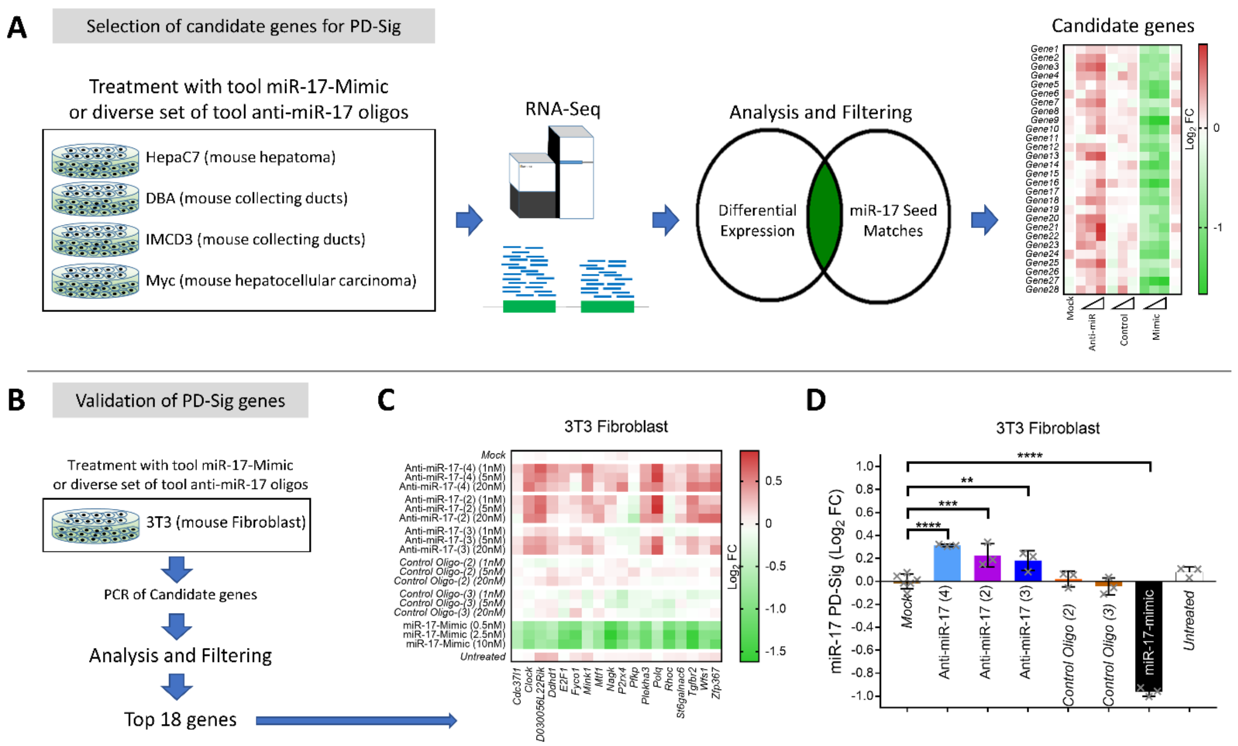

Supplementary Figure 2. ***Development of a mouse miR-17 PD-Signature (PD-Sig) for assessing miR-17 activity following anti-miR treatment.*** **(A)** Schematic illustration of the miR-17 PD-Sig candidate genes selection. Four different mouse kidney and non-kidney cell lines were transfected with tool anti-miR-17 oligos, a miR-17-mimic, or PBS for 24 h prior to RNA-seq analysis. Direct miR-17 target genes differentially regulated by treatments were ranked, filtered and selected. **(B)** Candidate genes were validated by qPCR in 3T3 cells following anti-miRs and mimic treatment. Following additional analysis and filtering, **(C)** top 18 ranked genes (*Plekha3, Rhoc, D030056l22rik, Clock, Polq, Mtf1, E2f1, Tgfbr2, P2rx4, Nagk, Mink1, Zfp367, Fyco1, Pfkp, Ddhd1, Cdc37l1, Wfs1,* and *St6galnac6*) were chosen. **(D)** The resultant miR-17 PD-Sig score is the average of the 18 genes' individual $\log_2$ FC (Fold Change; normalized by 6 housekeeping genes: *Tbp, Gusb, Rplp0, Hprt1, B2m,* and *Pak1ip1*) compared to mock control; and can be used to comprehensively and unbiasedly assess miR-17 activity to rank-order potency of anti-miR-17 or miR-17-mimic oligonucleotides following treatment. An example in 3T3 fibroblast cells transfected with mock (n=6), control oligos and anti-miRs at 20 nM and mimic at 10 nM was shown (n=3/treatment group). Shades of red indicate upregulation, while shades of green indicate downregulation of individual genes. **p<0.01, ***p<0.001, ****p<0.0001. One-way ANOVA, Dunnette's multiple comparison test. Error bars represent standard deviations.

Suppplementary Figure 3

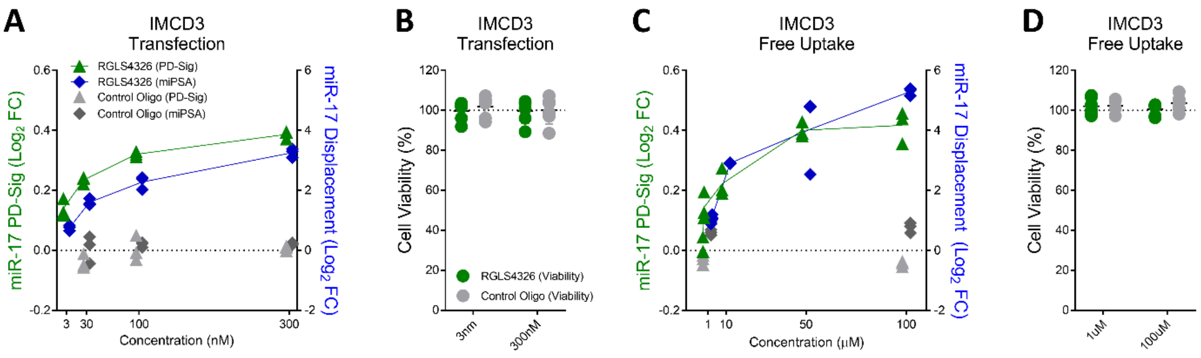

Supplementary Figure 3. ***RGLS4326 inhibits miR-17 activity but has no effect on cell viability in non-ADPKD kidney collecting duct cells.*** Concentration-dependent displacement of miR-17 (blue diamonds, as measured by miRNA polysome shift assay; miPSA; right y-axis) corresponded with concomitant de-repression of miR-17 PD-Sig (green triangles; left y-axis) in IMCD3 cells 24 h following RGLS4326 treatment by either **(A)** transfection or **(C)** free-uptake (n=3/group). **(B and D)** Inhibition of miR-17 following RGLS4326 treatment (green circle) has no effect on cell viability in IMCD3 cells 4 days following either transfection or free-uptake treatment (n=6). Control oligos (grey) was used at corresponding concentrations as negative control. Individual data points were shown.

Suppplementary Figure 4

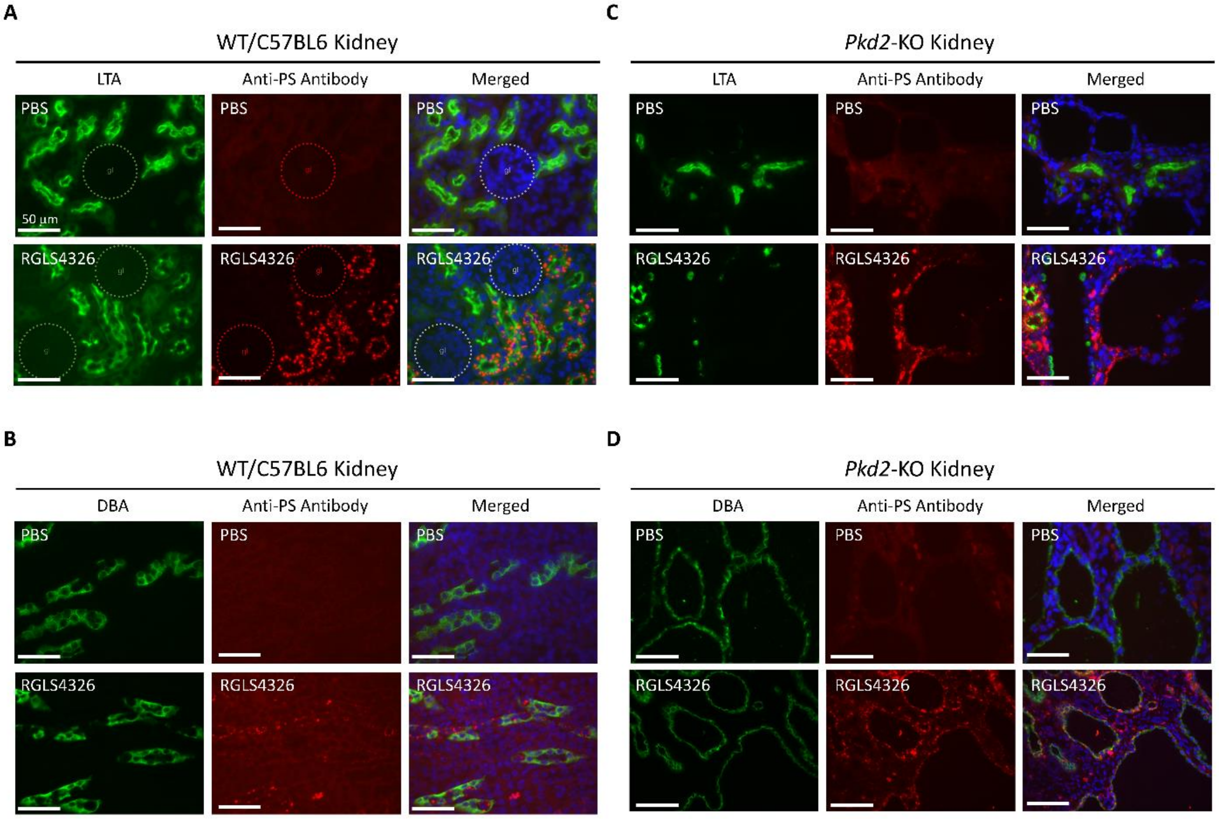

Supplementary Figure 4. ***Localization of RGLS4326 in proximal tubules and collecting duct cells following subcutaneous administrations.*** **(A-B)** WT/C57BL6 (n=3) or **(C-D)** *Pkd2*-KO mice (n=3) were dosed SC with PBS or 20 mg kg^{-1} of RGLS4326 on postnatal day (P)21, P22 and P23, and kidneys were harvested on P26. Kidney sections were co-stained with LTA (proximal tubules marker) or DBA (collecting ducts marker), anti-PS antibody (antibody labels RGLS4326) and DAPI. Representative immunofluorescence images of stained kidney sections demonstrating delivery of RGLS4326 to **(A and C)** proximal tubules and **(B and D)** collect duct cyst cells are shown. No glomerulus localization of RGLS4326 was observed in all mice tested. Glomerulus, *gl* (dotted circle). Scale bars, 50 µm.

Suppplementary Figure 5

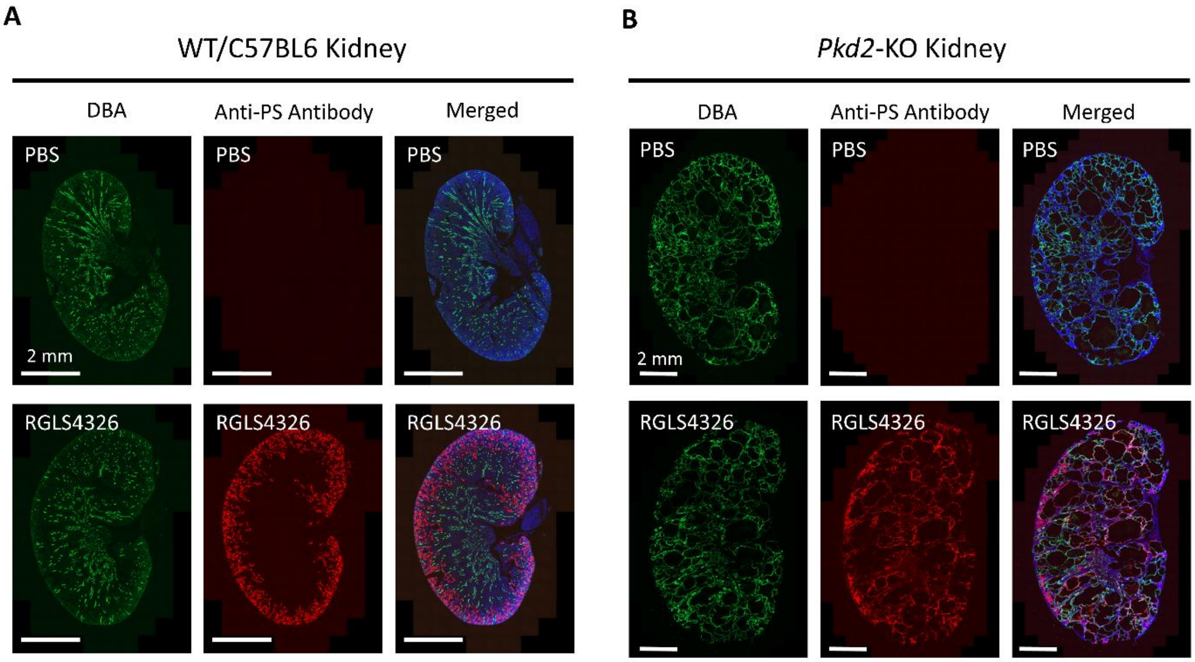

Supplementary Figure 5. ***Localization of RGLS4326 in normal and polycystic mouse kidney following subcutaneous administrations.*** **(A)** WT/C57BL6 (n=3) or **(B)** *Pkd2*-KO mice (n=3) were dosed SC with PBS or 20 mg kg^{-1} of RGLS4326 on postnatal day (P)21, P22 and P23, and kidneys were harvested on P26. Kidney sections were co-stained with DBA (collecting ducts marker), anti-PS antibody (antibody labels RGLS4326) and DAPI. Representative immunofluorescence images of stained kidney sections demonstrating delivery of RGLS4326 to cortical collecting ducts in normal mice and numerous collecting duct cysts in ADPKD mice are shown. Scale bars, 2 mm.

Suppplementary Figure 6

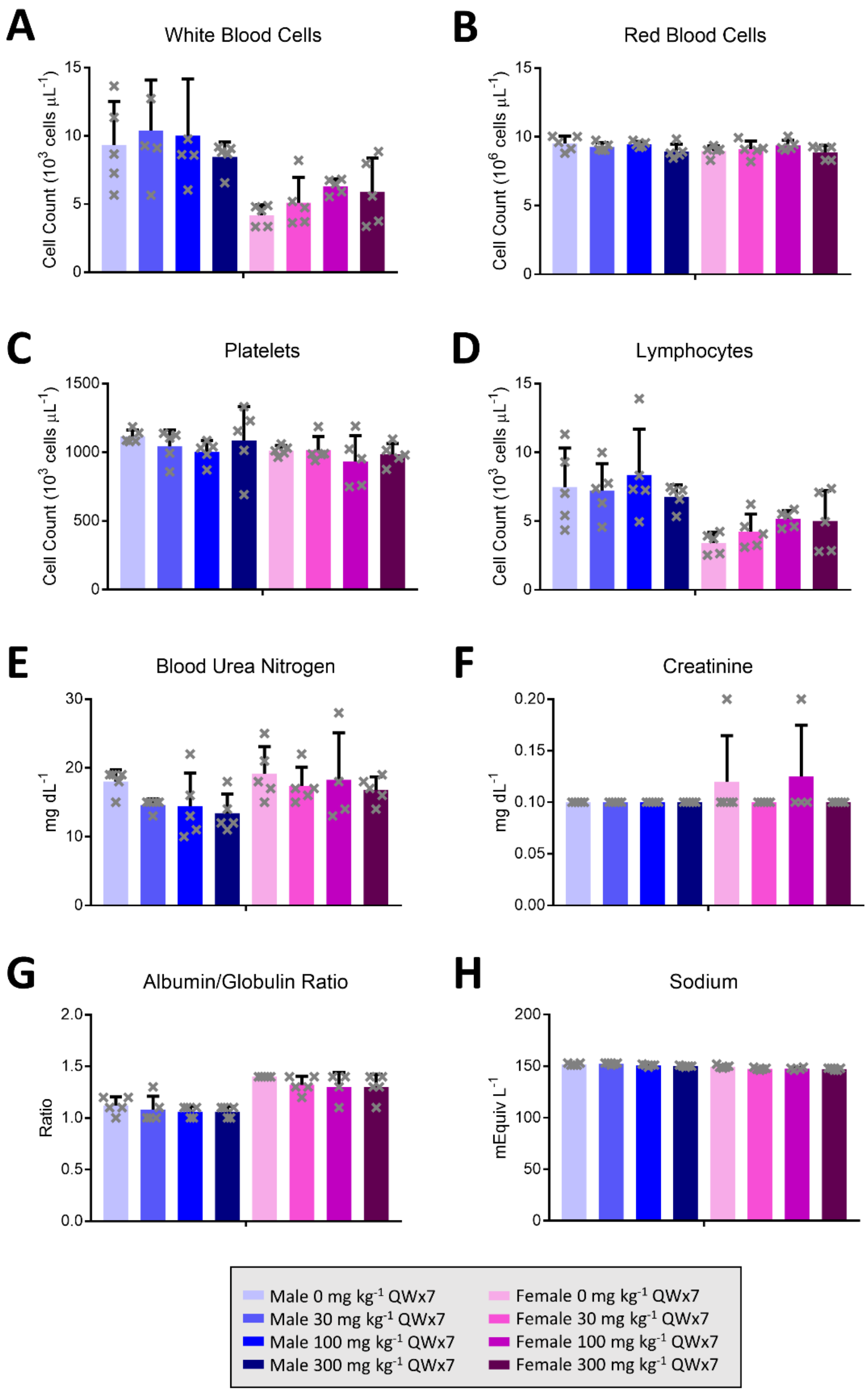

Supplementary Figure 6. ***Repeat dosing of RGLS4326 in mice has no effect on hematopoietic cell population and kidney function.*** CD1 mice (n=5/sex/group) were dosed SC QW with 0, 30, 100, or 300 mg kg^{-1} of RGLS4326 for 7 consecutive weeks (QWx7). Blood was collected for hematology and serum chemistry analysis at 48 h after the last dose. Specimens were analyzed using an Advia 120 automated hematology analyzer and an AU680 chemistry analyzer. Comparison between treatment groups indicated no hematological abnormalities **(A-D)** and no renal toxicity **(E-H)** following RGLS4326 treatment. Error bars represent standard deviations.

Suppplementary Figure 7

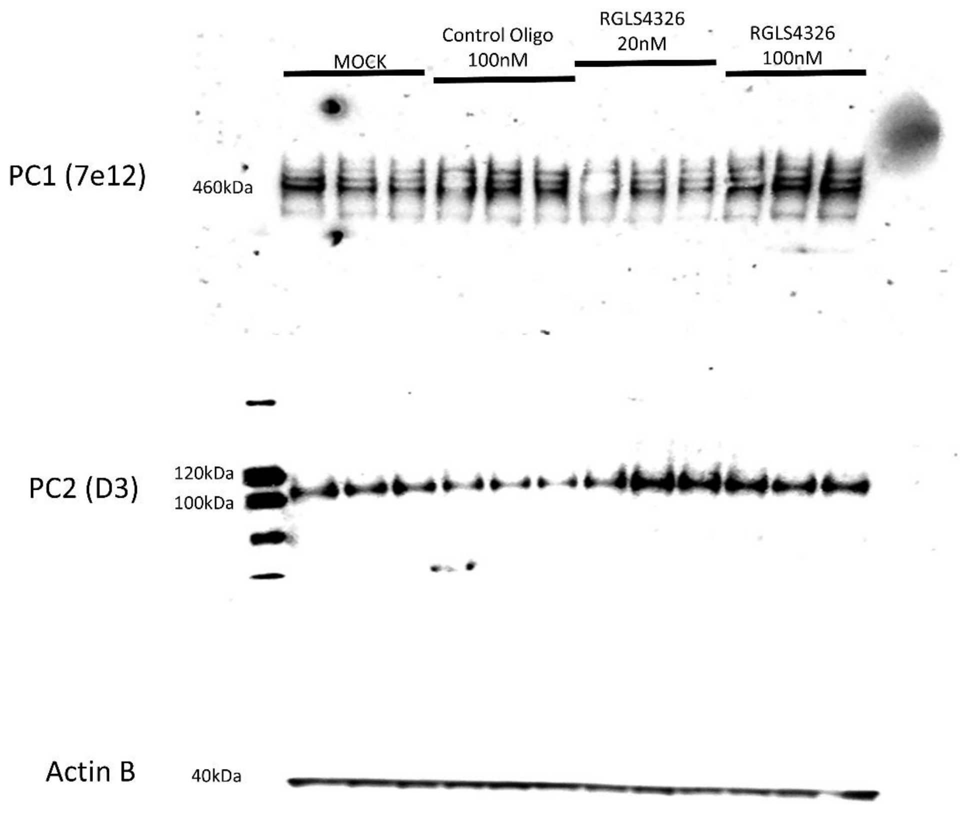

Supplementary Figure 7. ***The original Western blot demonstrating increased expression of PC1 and PC2 in primary ADPKD human cyst cells following RGLS4326 treatment.*** The blot was cut into three different sections (top, middle, bottom) prior to immunoblotting with respective primary antibody (PC1, PC2 and Actin-B, respectively). The blot is shown accompanied with indicated protein name (far left) and molecular weight (left).

Supplementary Table 1. ***Metabolic Stability Profile of RGLS4326***

In Vitro* Metabolic Stability (Mouse/Monkey/Human)	
Liver Lysate	98%/96%/98%
Kidney Lysate	100%/100%/100%
Whole Blood	100%/100%/100%
In Vivo* Metabolic Stability* (Mouse)	
Liver	>95%
Kidney	>95%
Plasma	>95%

*Percentage intact RGLS4326 following 24 h incubation at 5 μM in indicated mouse, monkey or human samples.

**Percentage intact RGLS4326 at 24 h following a single subcutaneous dose at 30 mg kg^{-1}.

Supplementary Table 2. ***Preclinical Safety Profile of RGLS4326***

**Genotoxicity**	
Bacterial Mutagenicity	Negative[&]
In Vitro Clastogenicity in Human Cells	Negative[&&]
In Vivo Clastogenicity in Mouse	Negative[&&&]
**Mitochondrial Toxicity**	
In Vitro Assessment in Human Cells	Negative[$]
In Vivo Assessment in Monkey	Negative[$$]
**Pro-inflammatory Liability**	
Ex Vivo Rat Tissue Slice Assay	Negative[#]
In Vivo Acute Mouse Study	Negative[##]

[&]Tested at up to 5000 μg $plate^{-1}$ in *S.typhymurium* and *E.coli.*

[&&]Tested at up to 500 μg mL^{-1} in human lymphocytes *in vitro.*

[&&&]Tested at maximum tolerated dose in CD1 mice in both micronucleus and comet assays *in vivo.*

[$]Tested at up to 100 μM in human HepG2 cells under glucose- or galactose-dependent conditions.

[$$]No evidence of treatment related changes in mitochondrial morphology and cellular density as examined by transmission electron microscopy in kidney, liver and heart samples following 6 weekly SC doses at 100 mg kg^{-1} in cynomolgus monkey.

[#]No significant changes in *Oas1a* and *Ifit3* expression after 24 h or 48 h incubation at 5 μM in Sprague Dawley rat liver or kidney slice culture, respectively.

[##]No significant changes in spleen weight per body weight ratios, serum ALT, AST, BUN, and creatinine, and kidney or liver *Oasl2* or *Ifit1* expression 96 h following a single subcutaneous dose at 300 mg kg^{-1} in SvJ129 mice.

Supplementary Table 3. ***List of miRNA probes***

**Gene**	**TaqMan microRNA Assay ID*<br>(Applied Biosystems)**
Hsa-miR-17	#002308
Hsa-Let-7d	#002283
U6 snRNA	#001973

*Sequence information proprietary to manufacturer (Applied Biosystems/Thermo Fisher Scientific). Please contact manufacturer and refer to specific Assay ID.

Supplementary Table 4. ***List of mRNA probes and primers***

Gene	PrimeTime Std qPCR Assay ID (IDT)	Probe Sequence (5'→3')	Primer 1 Sequence (5'→3')	Primer 2 Sequence (5'→3')
**Mouse miR-17 PD-Signature**				
Cdc37l1	Mm.PT.56a.9136085	6FAM/TCTTCAGTG/ZEN/TTGTACC CCTGCCC/3IABkFQ/	GTCCATCATTCGGTCATCATCT	GGCTCCTTAGAATCCTTACCAC
Clock	Mm.PT.58.6936121	6FAM/TCCAGTCCT/ZEN/GTCGAAT CTCACTAGCA/3IABkFQ/	ACTTCCATCTGTCATGATCGC	ACATAAAGAGACCACTGCACAG
D030056 L22Rik	Mm.PT.56a.11893193	6FAM/TGCACAGCC/ZEN/AGCCTGA AGAGTTT/3IABkFQ/	CAGATCAAGAGGTGGCAGAG	GGCGACAGAGTAGAAAGCG
E2f1	Mm.PT.58.32742399	6FAM/TCCAGCTCA/ZEN/TTGCCAA GAAGTCCA/3IABkFQ/	GCTTACCAATCCCCACCAT	GCGCATCTATGACATCACCA
Nagk	Mm.PT.58.6224614	6FAM/TCTCCCTGA/ZEN/TGTGCAC CTTCTGC/3IABkFQ/	CAGCCTTCCTGAAGATGTACC	TATTTCCAGGTGCCAGATCG
P2rx4	Mm.PT.58.33691452	6FAM/CCACACGAA/ZEN/CACCCA CCCAATGA/3IABkFQ/	CTGACCACAGAGTCCGTTTC	ATCCGCAGCCGTAAAGTG
Plekha3	Mm.PT.58.21748816	6FAM/CCGCCATCT/ZEN/TTATACT CCCTTTGCTCC/3IABkFQ/	CTCCATTCTTGTGTTGTCTGC	TCCTGTCCTACTATGACTCACA
Rhoc	Mm.PT.58.45919698	6FAM/CTGGTATGC/ZEN/TCATCTT GCCTCAGGT/3IABkFQ/	GCCTTCCTCAGATCGAACC	CGTGCCCATCATCCTAGTG
Tgfbr2	Mm.PT.58.6358355	6FAM/CCATCTGTG/ZEN/AGAAGCC GCATGAAGT/3IABkFQ/	AACCGTCTCCAGAGTAATGTTC	GACAACCAGAAGTCCTGCAT
Zfp367	Mm.PT.58.10557388	6FAM/TCATCTCCC/ZEN/AGTACTT TGCCAGCC/3IABkFQ/	CCTTCTGAACTAGCTTGCCTTT	CTGAAGAGAGAAGAGCCAACG
Polq	Mm.PT.58.5536034	6FAM/TGCCTTTCA/ZEN/AAAGTGC CCGGAAG/3IABkFQ/	GCCAGTCACCCAAATAGTTCT	GATAGTGCGGAGGTAGAGGT
Mtf1	Mm.PT.58.10188697	6FAM/CCACCAGTT/ZEN/CACCACA AAACCAAGAA/3IABkFQ/	CTGCTCCACTTTCGATGCTT	TGCAGTCCAGCCTAGTCAT
Mink1	Mm.PT.58.8539396	6FAM/TGACGTGCC/ZEN/GCCCCTT GTATAC/3IABkFQ/	ATCTCTTCCTCCTCATCCTCT	GTTGGCAATGGAACCTATGGA
Fyco1	Mm.PT.58.12062609	6FAM/CCAGTGCAA/ZEN/GGTCCTC ATTCCCA/3IABkFQ/	AAGATGAGCAAGTAGATGCCA	TTCCAAGAGACAGAAGACACG
Ddhd1	Mm.PT.58.6950944	6FAM/ATCAGCTTG/ZEN/GTTCCAG TACACCGG/3IABkFQ/	TCCCATCAATAAACCACTGTCC	TCTACGAGGTGGATGTCACT

Wfs1	Mm.PT.58.10459648	6FAM/CCTCGTCAG/ZEN/CAGTGAA TCCAAGAACT/3IABkFQ/	TCCACAAAATCGTCCAGAGC	GTCAACGAGCAGGATGGAG
St6galnac6	Mm.PT.58.41491160	6FAM/AAGTCCCAT/ZEN/TCCTGGT TGAGCACG/3IABkFQ/	CACACAATTCCACCGCAATC	CAGCAGTTTGACGACCTCTT
Pfkp	Mm.PT.58.23785960	6FAM/AAGCCGTGA/ZEN/AACTCC GAGGAAGG/3IABkFQ/	GACGCTTATAGGTGTTCAGGTT	AGTGTGTGCAAATGACCCA
Tbp	Mm.PT.39a.22214839	6FAM/ACTTGACCT/ZEN/AAAGACC ATTGCACTTCGT/3IABkFQ/	CCAGAACTGAAAATCAACGCA G	TGTATCTACCGTGAATCTTGGC
Gusb	Mm.PT.39a.22214848	6FAM/ACATCACCC/ZEN/AAGAAG CAGCCCT/3IABkFQ/	ACCACACCCAGCCAATAAAG	AGCAATGGTACCGGCAG
Rplp0	Mm.PT.58.43894205	6FAM/AGGCCCTGC/ZEN/ACTCTCG CTT/3IABkFQ/	CGCTTGTACCCATTGATGATG	TTATAACCCTGAAGTGCTCGAC
Hprt1	Mm.PT.39a.22214828	6FAM/CTTGCTGGT/ZEN/GAAAAGG ACCTCTCGAA/3IABkFQ/	AACAAAGTCTGGCCTGTATCC	CCCCAAAATGGTTAAGGTTGC
B2m	Mm.PT.39a.22214835	6FAM/CCGGAGAAT/ZEN/GGGAAG CCGAACATAC/3IABkFQ/	GGGTGGAACTGTGTTACGTAG	TGGTCTTTCTGGTGCTTGTC
Pak1ip1	Mm.PT.58.12717376	6FAM/ACTGACCAT/ZEN/GTCTTTT ACCCTGTTCTCATG/3IABkFQ/	TGCTGTGACAAGAACGTGAT	TGTGATTCCCTAGAGTGCCT
**Mouse or Rat Genes**				
Pkd1	Mm.PT.58.10660047	6FAM/ACACTCAAG/ZEN/GGACAC AATGGGGAC/3IABkFQ/	GGAACTTCGGCTCACTTCATAC	TGGAAAGCAGGTCGGAAG
Pkd2	Mm.PT.58.10481485	6FAM/CGAGACATG/ZEN/GTTGTGG AGAGCTGG/3IABkFQ/	ATTATGGTGAAGCCGAAGAGG	CTGTCATGGTATTTTTGGTCTGG
Lcn2 (Ngal)	Mm.PT.58.10167155	6FAM/TGTTCTGAT/ZEN/CCAGTAG CGACAGCC/3IABkFQ/	CCTGTGCATATTTCCCAGAGT	CTACAATGTCACCTCCATCCTG
Ifit1	Mm.PT.58.32674307	6FAM/ACAGCTACC/ZEN/ACCTTTA CAGCAACCAT/3IABkFQ/	TGAAGCAGATTCTCCATGACC	GCAAGAGAGCAGAGAGTCAAG
Oasl2	Mm.PT.56a.17167264	6FAM/CACTGCAGG/ZEN/TACCAG AACTTCACCA/3IABkFQ/	GCACCTCTTCCATATTTACGTC T	GCTTCACAGAGTTACAGAGACA
Ifit3	Rn.PT.58.13648367	6FAM/TCCAGAGAC/ZEN/TCCTTGT TGACCTCACT/3IABkFQ/	TCCATATGACTTGAGATGCTTC C	CATAGAAACAGATCGCCGTCA
Oas1a	Rn.PT.58.34651724	6FAM/TGCTTCCGA/ZEN/GATACTG TCCACCCA/3IABkFQ/	GTCTGACTTGCCCTTGAGTG	TCAGCTATCAATGTCCTGTGTG
**Human miR-17 PD-Signature**				
BTG3	Hs.PT.58.28207974	6FAM/AAACTCCTG/ZEN/ACCTCGT GATCCGC/3IABkFQ/	GCTCACGCCTGTAATCCA	ATGTGAGGTGTGCTGTCG
C7ORF43	Hs.PT.58.19749599	6FAM/TCTGCCACA/ZEN/CGCCCCT CA/3IABkFQ/	CCGGGAAAAGCCAAGGT	CTCCAAGACTTCCTTGCTGT

CROT	Hs.PT.58.20126844	6FAM/TCCATGATA/ZEN/AAATCACAATAGGAAACCAGTACACC/3IABkFQ/	GCAGTGATGGAAGAGAATCCT	GCAGTCTCAAGCAGTTCATCT
ENPP5	Hs.PT.58.19757218	6FAM/TGACTTTCT/ZEN/GTTAGGCAACCACGGT/3IABkFQ/	CTTTCTGAAGGCAGGACCAT	AACCAATCATAGCAGTGGCT
GUSB	Hs.PT.58v.27737538	6FAM/TGCAGGGTT/ZEN/TCACCAGGATCCAC/3IABkFQ/	GTTTTTGATCCAGACCCAGATG	GCCCATTATTCAGAGCGAGTA
HPRT1	Hs.PT.58v.45621572	6FAM/AGCCTAAGA/ZEN/TGAGAGTTCAAGTTGAGTTTGG/3IABkFQ/	GCGATGTCAATAGGACTCCAG	TTGTTGTAGGATATGCCCTTGA
LIMK1	Hs.PT.58.4056836	6FAM/CCAGCCCGA/ZEN/AGTCAGCCACC/3IABkFQ/	AGTCTTCTCGTCCACCATGA	GAACATCATCCACCGAGACC
MINK1	Hs.PT.58.3502144	6FAM/CACCGCAAC/ZEN/ATCGCCACCTACT/3IABkFQ/	CCACAGAACTCCATCACCAG	GAGGAAGAGATCAAACAGGAGA
NAGK	Hs.PT.58.21111037	6FAM/ATTGGACTC/ZEN/CCCATCCTGTGCG/3IABkFQ/	AAACCTTCCTTCAGCAGCTC	GCAGACACATCGTAGCAGT
NKIRAS1	Hs.PT.58.39278922	6FAM/CAGCTCCTT/ZEN/TATGGAAATCATACTATTGGAATGGA/3IABkFQ/	TCGGTCTGTTTCTACTGAAGC	GGTTTGTGGATTGTTATCTGTGG
PLEKHA3	Hs.PT.56a.19457926	6FAM/TCCTCTTGA/ZEN/AGACCCAGATAGACCTGTTC/3IABkFQ/	GCTTTCTTCAGGAATGGTTGC	GCCGAAGAACCTACTCAGATAC
PTPN4	Hs.PT.58.22922591	6FAM/TGCTGACCT/ZEN/CTGTGTCCCTAGACTG/3IABkFQ/	CATGAGTGTGTTCTGCAATGTC	CTGTGATTGTGTCTCGAGTAGC
RPLP0	Hs.PT.39a.22214824	6FAM/CCCTGTCTT/ZEN/CCCTGGGCATCAC/3IABkFQ/	TGTCTGCTCCCACAATGAAAC	TCGTCTTTAAACCCTGCGTG
TBC1D9	Hs.PT.58.19975995	6FAM/TCAAGCCAT/ZEN/TCCCAGTGTTCAGTGAT/3IABkFQ/	GGAGAGTGTCTGCAAGAGAT	GCTCCTTACCGAATCTTGTACC
B2M	Hs.PT.58v.18759587	6FAM/CCTGCCGTG/ZEN/TGAACCATGTGACT/3IABkFQ/	ACCTCCATGATGCTGCTTAC	GGACTGGTCTTTCTATCTCTTGT
TBP	Hs.PT.58v.39858774	6FAM/TGATCTTTG/ZEN/CAGTGACCCAGCATCA/3IABkFQ/	CAGCAACTTCCTCAATTCCTTG	GCTGTTTAACTTCGCTTCCG
TGFBR2	Hs.PT.58.2243027	6FAM/TTCTCCATA/ZEN/CAGCCACACAGACTTCC/3IABkFQ/	CTGTCTCTAGTGTTATGTTCTCGTC	TGACAACCAGAAATCCTGCAT

6FAM, 5’-6-FAM™ 520nm Fluorescein; **ZEN**, Internal ZEN™ quencher; **3IABkFQ**, 3’-Iowa Black® FQ quencher.
