# Supplementary material for: Discovery and preclinical evaluation of anti-miR-17 oligonucleotide RGLS4326 for the treatment of polycystic kidney disease
Source: Nat Commun. 2019 Sep 12;10:4148. doi: 10.1038/s41467-019-11918-y (PMC6742637; doi:10.1038/s41467-019-11918-y)
Supplement: Supplementary file 2 — Reporting Summary [file 41467_2019_11918_MOESM2_ESM.pdf]

nature research

Corresponding author(s): Edmund Lee, PhD
Vishal Patel, MD

Last updated by author(s): Jul 18, 2019

Reporting Summary

Nature Research wishes to improve the reproducibility of the work that we publish. This form provides structure for consistency and transparency in reporting. For further information on Nature Research policies, see Authors & Referees and the Editorial Policy Checklist.

Statistics

For all statistical analyses, confirm that the following items are present in the figure legend, table legend, main text, or Methods section.

n/a	Confirmed	
☐	☒	The exact sample size (*n*) for each experimental group/condition, given as a discrete number and unit of measurement
☐	☒	A statement on whether measurements were taken from distinct samples or whether the same sample was measured repeatedly
☐	☒	The statistical test(s) used AND whether they are one- or two-sided<br>*Only common tests should be described solely by name; describe more complex techniques in the Methods section.*
☐	☒	A description of all covariates tested
☐	☒	A description of any assumptions or corrections, such as tests of normality and adjustment for multiple comparisons
☐	☒	A full description of the statistical parameters including central tendency (e.g. means) or other basic estimates (e.g. regression coefficient) AND variation (e.g. standard deviation) or associated estimates of uncertainty (e.g. confidence intervals)
☐	☒	For null hypothesis testing, the test statistic (e.g. *F*, *t*, *r*) with confidence intervals, effect sizes, degrees of freedom and *P* value noted<br>*Give P values as exact values whenever suitable.*
☒	☐	For Bayesian analysis, information on the choice of priors and Markov chain Monte Carlo settings
☒	☐	For hierarchical and complex designs, identification of the appropriate level for tests and full reporting of outcomes
☐	☒	Estimates of effect sizes (e.g. Cohen's *d*, Pearson's *r*), indicating how they were calculated

Our web collection on statistics for biologists contains articles on many of the points above.

Software and code

Policy information about availability of computer code

Data collection	Data was collected using the NextSeq 500 sequencer per manufacturer's protocol (Illumina).
Data analysis	Sequencing quality was assessed with the FastQC tool v0.11.2 and trimmed with TrimGalore. Reads were mapped using STAR with samtools v1.1. HTSeq v0.6.1 was used to generate counts. Map and count quality was assessed by picard tools v1.119 and RSeQC tool. Differential expression was performed using edgeR v3.20.2 in R version 3.4.2. Ingenuity Pathway Analysis software was used to predict putative gene networks involved with input differential mRNA expression data.

For manuscripts utilizing custom algorithms or software that are central to the research but not yet described in published literature, software must be made available to editors/reviewers. We strongly encourage code deposition in a community repository (e.g. GitHub). See the Nature Research guidelines for submitting code & software for further information.

Data

Policy information about availability of data

All manuscripts must include a data availability statement. This statement should provide the following information, where applicable:

- Accession codes, unique identifiers, or web links for publicly available datasets
- A list of figures that have associated raw data
- A description of any restrictions on data availability

All data analysed during this study are included in this published article (and its supplementary information files).

Field-specific reporting

Please select the one below that is the best fit for your research. If you are not sure, read the appropriate sections before making your selection.

☒ Life sciences ☐ Behavioural & social sciences ☐ Ecological, evolutionary & environmental sciences

For a reference copy of the document with all sections, see nature.com/documents/nr-reporting-summary-flat.pdf

Life sciences study design

All studies must disclose on these points even when the disclosure is negative.

Sample size	For in vitro experiments 3-7 replicates were included for each condition. For pharmacokinetic and pharmacodynamic experiments 3-5 animals were included for each condition based on variability of endpoints to be measured. For quantitative whole body radiography experiments, only a single animal was used for each condition. For in vivo efficacy experiments 12-15 animals were used in each arm of the study based on the variability of disease progression for each model from previous publications.
Data exclusions	No data were excluded
Replication	All in vitro experiments were repeated at least 2 times.
Randomization	For efficacy study, Pkd2KO mice from each litter were randomized across treatment groups until full enrollment of the study. For Pcy/CD1 and Pcy/DBA mice, age-matched animals were randomized based on similar initial body weights across treatment groups. For MRI-based efficacy study, animals were randomized based on similar initial bwTKV across treatment groups.
Blinding	For Pkd2KO efficacy study, investigators were blinded to treatment group until predetermined analysis was completed.

Reporting for specific materials, systems and methods

We require information from authors about some types of materials, experimental systems and methods used in many studies. Here, indicate whether each material, system or method listed is relevant to your study. If you are not sure if a list item applies to your research, read the appropriate section before selecting a response.

Materials & experimental systems

n/a	Involved in the study	
☐	☒	Antibodies
☐	☒	Eukaryotic cell lines
☒	☐	Palaeontology
☐	☒	Animals and other organisms
☒	☐	Human research participants
☒	☐	Clinical data

Methods

n/a	Involved in the study	
☒	☐	ChIP-seq
☒	☐	Flow cytometry
☒	☐	MRI-based neuroimaging

Antibodies

Antibodies used	For IF: Anti-phosphohistone H3 (1:400, Sigma-Aldrich H0412) and Anti-PS (1:1000, Regulus Therapeutics). Secondary antibodies were conjugated to Alexa Fluor 488 (Molecular Probes).<br>For WB: Anti-polycystin-1 (1:200, Santa Cruz 7E12), Anti-polycystin-2 (1:200, Santa Cruz D3) and Anti-Actin-B (1:5000, Invitrogen BA3R)
Validation	Anti-pH3 validated for immunofluorescence using human HeLa cell line treated with nocodazole (Manufacturer), Anti-PS validated for immunohistochemistry using tissue samples from mice dosed with PS-oligos (Regulus)

Eukaryotic cell lines

Policy information about cell lines

Cell line source(s)	IMCD3, M1, MDCT, NIH/3T3, Hep-C7 and HeLa were obtained from ATCC. DBA-WT, DBA-PKD, LTL-WT and LTL-WT were obtained from Discovery BioMed. Myc-HCC were obtained from Dr. Dean Felsher Lab.
Authentication	Performed on selected cell lines
Mycoplasma contamination	Performed on selected cell lines

Commonly misidentified lines (See ICLAC register)	No commonly misidentified cell lines were used

Animals and other organisms

Policy information about studies involving animals; ARRIVE guidelines recommended for reporting animal research

Laboratory animals	Pkd2KO mice were generated from Pkhd1/Cre and Pkd2(F/F) as previously described (ref 17); JCK/C57BL6 and Pcy/CD1 mice were obtained from CrownBio; Pcy/DBA mice were obtained from Kyudo Co; 129X1/SvJ, WT/C57BL6 and WT/DBA mice were obtained from Jackson Laboratory. WT/CD1 mice were obtained from Charles River and SNBL-USA. Sprague-Dawley rats were obtained from Charles River. Cynomolgus Monkeys were obtained from SNBL-USA.
Wild animals	The study did not involve wild animals
Field-collected samples	The study did not involve samples collected from the field
Ethics oversight	All animal experiments were conducted in accordance with the institutional AAALAC Guidelines and approved by the Institutional Animal Care and Use Committees of Explora, UT Southwestern, and SNBL-USA where experiments were performed

Note that full information on the approval of the study protocol must also be provided in the manuscript.
